# Strategically isolated bacteriophages targeting ETEC K88 (F4) alleviate post-weaning diarrhea in piglets via modulation of gut microbiota and inflammatory responses

**DOI:** 10.1186/s40104-025-01322-6

**Published:** 2026-01-18

**Authors:** Yan Chen, Minfeng Ding, Xingping Chen, Tiande Zou, Yi Liu, Jun Chen, Jinming You

**Affiliations:** 1 Jiangxi Province Key Laboratory of Animal Nutrition and Feed, Jiangxi Agricultural University, Nanchang, 330045 China; 2 College of Animal Science and Technology, Jiangxi Agricultural University, Nanchang, 330045 China

**Keywords:** Bacteriophages, ETEC K88, Gut microbiota, Inflammation, Post-weaning diarrhea, Weaned piglets

## Abstract

**Background:**

Post-weaning diarrhea (PWD) in piglets, primarily caused by enterotoxigenic *Escherichia coli* (ETEC) K88 (F4) infection, presents a major challenge in swine production. This study aimed to isolate bacteriophages (phages) specific to ETEC K88, utilizing ETEC K88 as the host strain, and to assess the efficacy of dietary supplementation with the isolated phages in weaned piglets over a two-week period using an ETEC K88 challenge model in a pilot study.

**Results:**

Three ETEC K88-specific phages (EC-P1, EC-P2, and EC-P3) were isolated and identified as tailed phages. These phages displayed a short latency period, broad acid–base stability, and thermal stability, effectively inhibiting ETEC K88 growth and disrupting ETEC K88 biofilms in vitro. Lyophilized phage powder was prepared and supplemented at 400, 600 or 800 mg/kg in the diets. Compared to the ETEC K88 group, piglets in the ETEC K88 + 600 or 800 mg/kg phages group exhibited markedly lower diarrhea scores and rectal temperatures at 12, 24, and 48 h post-infection. Supplementation with 600 mg/kg phages enhanced intestinal integrity of ETEC K88-infected piglets, as evidenced by an increased jejunal villus height and villus height-to-crypt depth ratio, reduced serum diamine oxidase and D-lactate levels, and upregulated jejunal ZO-1 protein expression. Concomitantly, systemic and jejunal inflammatory responses were attenuated by supplementation with 600 mg/kg of phages, as evidenced by decreased serum LPS, IL-1β, IL-10 and TNF-α levels, down-regulated jejunal *IL-1β* and *IL-6* mRNA expression, and suppressed NF-κB signalling (downregulated p-IκBα/IκBα and p-p65/p65 ratios). Supplementation with 600 mg/kg phages also shifted the faecal microbiota toward eubiosis, increasing the Shannon index, decreasing Proteobacteria and Enterobacteriaceae abundances, and elevating beneficial taxa (Patescibacteria, Muribaculaceae, and *Subdoligranulum*). Correlation analysis further revealed that Proteobacteria and Enterobacteriaceae abundances were positively associated with diarrhoea characteristics, whereas Muribaculaceae showed a negative correlation.

**Conclusions:**

Three ETEC K88-targeting phages were successfully isolated, characterized, and prepared as lyophilized phage powder for dietary supplementation. Dietary supplementation with 600 mg/kg of lyophilized phage powder alleviated PWD in piglets by modulating gut microbiota and inflammatory responses.

**Supplementary Information:**

The online version contains supplementary material available at 10.1186/s40104-025-01322-6.

## Background

Post-weaning diarrhea (PWD) in piglets is a prevalent and serious threat in global swine production, leading to high mortality rates and substantial economic losses for swine industry [1, 2]. PWD in piglets typically occurs within the first two weeks after weaning [2]. During this critical period, enterotoxigenic *Escherichia coli* (ETEC) K88 (F4) is widely recognized as the primary causative pathogen [3, 4]. The use of in-feed antibiotics has been widely implemented as an effective strategy to reduce the incidence of PWD and enhance the growth performance of piglets [5]. However, in-feed antibiotics has been prohibited in multiple nations due to concerns regarding the development of antibiotic-resistant bacteria and the detection of antibiotic residues [6]. Therefore, it is urgent to explore potential anti-pathogenic agents for controlling PWD in piglets, particularly given the prohibition of in-feed antibiotics in numerous countries and regions.

Bacteriophages (also known as phages) have been utilized for their antimicrobial effects against bacterial infections since their discovery in the early 1900 s [7]. Nevertheless, their therapeutic use declined with the advent of broad-spectrum antibiotics [7]. The rise of antibiotic resistance has reignited international interest in phage-based therapies for treating pathogenic bacteria infections [6, 8]. Compared to antibiotics, phages have advantages such as targeted efficacy, bacterial elimination, and proliferation against pathogenic bacteria, even at low concentrations [9]. Importantly, phages can mitigate the development of antibiotic-resistant bacterial strains and avoid the disruption of microbial homeostasis associated with antibiotic use [9, 10]. Our previous study found that incorporating a phage cocktail as a dietary alternative to antibiotics enhanced growth performance in newly weaned piglets by modulating intestinal inflammation, enhancing intestinal barrier integrity, and regulating gut microbiome [11]. Additionally, it has been demonstrated that phages exhibit considerable potential in the treatment of bacterial infections associated with gastrointestinal diseases [12]. In another of our study utilizing an enterotoxigenic *Escherichia coli* (ETEC)-challenged weaned mouse model, we observed that dietary supplementation with a phage cocktail alleviated ETEC-induced diarrhea and intestinal injury by modulating intestinal inflammation and gut microbiome [13]. It should be noted that strategically isolating phages using specific pathogenic bacteria as host strains is a highly effective approach for obtaining phages tailored to target specific pathogens [6]. As such, targeted isolation of ETEC K88-specific phages could serve as a feasible strategy for controlling PWD in piglets, given that ETEC K88 is a primary causative agent of PWD.

Therefore, this study aimed to isolate ETEC K88-targeting phages using ETEC K88 as the host bacterium and evaluate the potential of dietary supplementation with these phages in piglets during the two-week post-weaning period using an ETEC K88 challenge model.

## Materials and methods

### Isolation and purification of phages

For phage isolation, sewage samples were collected from 10 commercial pig farms in Jiangxi Province, China. A standard strain of ETEC K88 (O141:K85, K88ab, the National Center for Veterinary Culture Collection, Beijing, China) [14] was selected as the host bacterium for phage isolation using the double-layer plate method [15]. This strain can produce heat-stable (ST) toxin and heat-labile (LT) toxin [16]. Enterotoxin-producing genes were also measured in this strain, confirming the presence of ST (*estA* and *estB* genes) and LT (*elt-Ⅰ* gene) toxins (Fig. S1 in Additional file 1). The sewage supernatant was collected by centrifugation and filtered through a 0.22-μm membrane to remove bacterial cells. Subsequently, ETEC K88 was added and incubated at 37 °C for 12 h to propagate phages. The mixture was then filtered through a 0.22-μm membrane to obtain the phage stock solution. The phage stock solution was mixed with the host bacterial suspension in 5 mL of Luria-Bertani (LB) medium (containing 0.5% agar) and immediately overlaid onto the bottom agar medium. After solidification, the plates were incubated at 37 °C to obtain plaques. The plaque-forming phages were purified 3–5 times until uniform morphology and size were observed, yielding the purified phage. Finally, the phage morphology was examined using transmission electron microscopy.

### Whole genome sequencing of isolated phages

DNA was extracted from isolated phages using a viral DNA kit (D6900-01, Omega Bio-tek, Norcross, GA, United States). Upon receiving the DNA sample, a sequencing library was prepared from the tested samples. After obtaining sequencing data for each sample, data quality was assessed, and low-quality reads were filtered to ensure the reliability of downstream analysis. Quality control was performed using Soapnuke software. Clean reads were aligned to the host genome using BWA software, and host sequences were removed. High-quality reads from each sample were assembled into contigs using Megahit software. The assembled contigs were compared against the CheckV virus database to identify viral sequences, and sequencing depth was calculated using samtools depth. Gene prediction on contigs was performed with Prokka software, and the number and length of predicted genes were quantified. Data processing and analysis were conducted by Guangdong Magigene Biotechnology Co., Ltd. (Guangzhou, China).

### Biological characterization of isolated phages

#### Host range

The host range of three isolated phages (EC-P1, EC-P2, and EC-P3) was evaluated to assess their lytic activity against common gut pathogenic bacteria, including six enterotoxigenic *Escherichia coli* strains (CVCC 195, CVCC 199, CVCC 200, CICC 10413, CICC 10414, and CICC 10415), three enteropathogenic *E. coli* strains (CICC 10372, CICC 10411, and CICC 10412), one enterohemorrhagic *E. coli* strain (CICC 21530), two *Salmonella typhimurium* strains (CICC 21483), and one *Staphylococcus aureus* strain (CICC 21600). These strains were purchased from the National Center for Veterinary Culture Collection (CVCC, Beijing, China) or the China Center of Industrial Culture Collection (CICC, Beijing, China). A volume of 100 μL of bacterial suspension from each strain was transferred to a 10-mL centrifuge tube, mixed with 4 mL of top agar, and poured onto a prepared base agar plate. Subsequently, 5 μL of each phage culture was spotted onto the plate, which was then inverted and incubated at 37 °C for 8–10 h. Following incubation, plaque formation in the spotted regions was assessed. Clear plaques, indicating bacterial lysis, were recorded as “ + ”. The absence of plaques, indicating no lysis, was recorded as “–”.

#### Optimal multiplicity of infection

The optimal multiplicity of infection (MOI) for the three isolated phages (EC-P1, EC-P2, and EC-P3) was determined following the method described by Zhou et al. [17], with minor modifications. Briefly, specific MOI values (0.00001, 0.0001, 0.001, 0.01, 0.1, 1, and 10) were tested by mixing 100 μL of phage suspension with 900 μL of host bacteria ETEC L88 in 4 mL of LB liquid medium. The mixture was incubated at 37 °C with shaking at 180 r/min for 6 h. Phage titers were then quantified using the double-layer plate method. The optimal MOI was defined as the value yielding the highest phage titer. Phage titer (PFU/mL) was calculated as follows: plaque count × dilution factor × 100. All assays were performed in triplicate.

#### One-step growth curve assay

The one-step growth curves of three phages were determined according to the method of Shende et al. [18] with slight modifications. Phages EC-P1, EC-P2, or EC-P3 were mixed with host bacteria ETEC K88 at their optimal MOI in LB liquid medium, respectively. The mixture was incubated at 37 °C with shaking at 180 r/min for 150 min. During incubation, the growing bacterial cultures were collected at 10-min intervals, and phage titers were measured using the double-layer plate method. All assays were performed in triplicate.

#### Thermal stability and acid–base stability

For the thermal stability assay, the phage suspension was incubated at various temperatures (4, 10, 20, 30, 40, 50, 60, and 70 °C) for durations of 5, 10, 20, 40, and 60 min at pH 7.0. Phage titers were subsequently quantified using the double-layer agar plate method [19]. To assess acid-base stability, the phage suspension was incubated at 37 °C across a pH range of 3.0–14.0 for 1 h, followed by titer determination. All assays were performed in triplicate.

#### Evaluation of in vitro bacteriostatic activity

To assess antibacterial activity, the isolated phages EC-P1, EC-P2, or EC-P3 were each mixed with 1 mL of host bacteria ETEC K88 in 100 mL of LB liquid medium. The mixture was then incubated at 37 °C and 180 r/min, with the OD_600_ value measured hourly with a microplate reader (Tecan, Infinite M200 Pro, Switzerland). The biofilm removal assay was performed following the method described by Kher et al. [20]. Briefly, 200 µL of host bacterial suspension was added to each well of a 96-well plate and incubated at 37 °C for 48 h. The bacterial suspension was then dispensed and washed with sterile phosphate-buffered saline (PBS). Subsequently, 200 µL of phage solution (phage groups) or PBS (control group) was added and incubated for 24 h. Following incubation, the mixture in each well was washed with PBS and stained with 1% crystal violet for 30 min. The 96-well plate was then rinsed with sterile PBS, dried, destained with acetic acid, and the absorbance was measured at 590 nm with a microplate reader (Tecan, Infinite M200 Pro, Switzerland).

### Preparation of lyophilized powder of isolated phages for dietary supplementation

After adjusting the concentrations of each of the three phages to 1.0 × 10^9^ CFU/mL, the phage solution was centrifuged, and the supernatant was collected and sterilized by filtration through a 0.22-μm membrane. The suspensions of the three phages were mixed in equal volumes, with 20% skim milk and 0.1 mol/L trehalose added as protective agents. Following thorough homogenization, the mixture was freeze-dried in a lyophilizer (SCIENTZ-10N, SCIENTZ, Ningbo, China). The resulting phage lyophilizates were tested, confirming a phage concentration of 1.0 × 10^8^ CFU/g for each phage.

### Animal experimental design

The animal protocol was approved by the Animal Care and Use Committee of Jiangxi Agricultural University (Ethics Approval Number: JXAULL-2024-823). A total of 30 weaned barrows aged 21 d (Duroc × Landrace × Yorkshire; average body weight: 6.85 ± 0.75 kg) were assigned by body weight to five treatment groups, with six replicates and one piglet per replicate in a pilot study. The 30 healthy male piglets were selected from six sows (Landrace × Yorkshire) inseminated with semen from a Duroc boar via artificial insemination. The piglets were equally distributed, ensuring that each litter (five piglets per litter) from every sow was represented across the five treatment groups. The Duroc × Landrace × Yorkshire (DLY) piglets are a commercial breed widely utilized in swine production and have been demonstrated to be susceptible to ETEC F4ab using the genetic marker CHCF1 in a previous study [21]. The five treatment groups included the control group (basal diet), the ETEC K88 group (basal diet with oral sterile normal saline), and the ETEC K88 + phage groups (basal diet supplemented with 400, 600, or 800 mg/kg phages and challenged with ETEC K88). As such, the estimated PFU per gram of feed in the three phage-supplemented diets was 1.2 × 10^5^, 1.8 × 10^5^, and 2.4 × 10^5^ PFU/g, respectively (0.4 × 10^5^, 0.6 × 10^5^, and 0.8 × 10^5^ PFU/g for each of the three phages in the respective diets). The piglets were housed in individual cage with one piglet per cage. The individual housing of piglets was also implemented for ETEC-challenged piglets, as demonstrated in previous studies [22, 23]. The basal diet was formulated to meet the nutrient requirement of piglets recommeded by NRC (2012) [24] (Table 1). The experiment lasted for 14 d, in which the piglets were orally adminstrated with ETEC K88 (1 × 10^10^ CFU/mL, 10 mL/pig) or sterile normal saline (10 mL/pig) on d 12. Prior to the ETEC K88 challenge, the piglets were checked for diarrheal conditions, and the piglets were confirmed without clinical diarrhea symptoms. All piglets had free access to feed and fresh water throughout the whole experiment.

**Table 1:** Ingredient composition and nutritional levels of the basal diet (as fed basis)

Item	Ingredient, %	Item	Nutrient levels^b^
Corn	18.59	Digestible energy, kcal/kg	3,595.00
Extruded corn	29.00	Crude protein, %	19.10
Dehulled soybean meal	3.00	SID lysine, %	1.33
Whey powder	7.50	SID methionin, %	0.39
Fish meal	5.00	SID threonine, %	0.78
Soy protein concentrate	5.00	SID tryptophan, %	0.24
Wheat flour	10.00	Calcium, %	0.81
Extruded soybean	17.00	Total phosphorus, %	0.68
Soybean oil	1.25	Available phosphorus, %	0.42
Dicalcium phosphate	1.00		
Limestone	0.70		
Salt	0.10		
Choline chloride (60%)	0.05		
L-Lysine hydrochloride	0.80		
DL-Methionine	0.16		
L-Threonine	0.32		
L-Tryptophan	0.10		
Vitamin and mineral premix^a^	0.13		
Zeolite powder	0.30		
Total	100.00		

^a^The vitamin and mineral premix offers the subsequent values per kilogram of diet: vitamin A 10,000 IU, vitamin D_3_ 1,500 IU, vitamin E 50 IU, vitamin K_3_ 2.5 mg, vitamin B_1_ 4.5 mg, vitamin B_2_ 12 mg, vitamin B_6_ 10 mg, vitamin B_12_ 60 μg, niacin 60 mg, pantothenic acid 36 mg, folic acid 1 mg, biotin 0.5 mg, iron 100 mg, copper 6 mg, manganese 4 mg, zinc 100 mg, selenium 0.3 mg, and iodine 0.14 mg

^b^Calculated value

### Data record and sample collection

The body weight of each piglet was measured on d 1 and d 14, and individual feed intake was recorded on a weekly basis by weighing the provided feed and refusals. The average daily gain (ADG), average daily feed intake (ADFI), and feed conversion ratio (FCR) of the pigs were calculated accordingly. Additionally, the diarrhea score and rectal temperature of each piglet were assessed at 12, 24, and 48 h post-infection with ETEC K88. The fecal scoring criteria followed the method described by Deng et al. [25]. The feces of piglets were sampled at 48 h post-infection with ETEC K88 for subsequent analysis dry matter content and ETEC K88 load.

On d 15, blood, jejunal tissues, and fecal samples were collected from all piglets in the CON group, ETEC K88 group, and ETEC K88 + 600 mg/kg phages group (selected based on growth performance and diarrhea characteristics). Fresh fecal samples were collected in sterile tubes, immediately frozen in liquid nitrogen, and stored for subsequent fecal microbiota and short-chain fatty acid analysis. Blood was sampled from the superior vena cava of piglets, and serum was harvested via centrifugation at 3,000 r/min for 15 min, then stored at −80 °C for subsequent serum parameter analysis. After blood collection, the 18 piglets were slaughtered, and the entire jejunum was separated. The middle section of the jejunum was selected for sample collection. Approximately 2 cm of the jejunum was fixed in 4% paraformaldehyde solution for morphological analysis. Additionally, the jejunal mucosa was gently scraped using a microscope slide and stored at −80 °C for RT-qPCR and Western blot analysis.

### Analysis of fecal ETEC K88 load and fecal dry matter content

Fecal ETEC K88 load was detected using real-time fluorescent quantitative PCR as described by in previous studies [26, 27]. Fecal DNA was extracted using a fecal DNA extraction kit (AG21036, Accurate, Changsha, China). The recombinant plasmid containing the *estA* gene fragment was used as the standard. The primers used were: estA-pUC19-F: aaaacgacggccagtgaattcGAAACAACATGACGGGAGGTAAC; estA-pUC19-R: gaccatgattacgccaagcttGCACAGGCAGGATTACAACAAA (GenBank: KY581592.1). A standard curve was plotted by correlating the cycle threshold values of each sample with the logarithm of the target gene copy number. Fecal dry matter content was measured according to the AOAC method [28], which was also employed for dry matter content analysis in feces of piglets in our previous study [29].

### Serum parameter measurement

The serum parameters, including D-lactate (kit number: m1360341), diamine oxidase (DAO; m1002413), lipopolysaccharide (LPS; m196956), interleukin-1β (IL-1β) (m1025973), interleukin-6 (IL-6; m1025981), interleukin-10 (IL-10; m1025956), and tumor necrosis factor-α (TNF-α; m1002360), were measured using commercial enzyme-linked immunosorbent assay kits (Shanghai Enzyme-linked Biotechnology Co., Ltd., Shanghai, China) in accordance with the manufacturer’s protocols.

### Intestinal morphology analysis

The jejunal tissues were removed from the 4% paraformaldehyde solution for morphological analysis, as described in our previous study [30, 31]. Briefly, the tissues were dehydrated through a graded ethanol series and embedded in paraffin. Cross-sections of the jejunal tissues were prepared and stained with hematoxylin and eosin (H&E). The stained sections were examined using an EVOS microscope (Advanced Microscopy Group, Bothell, USA). Villus height and crypt depth were measured using Image-Pro Plus 6.0 software (Media Cybernetics, Rockville, USA), and the villus height-to-crypt depth (VH/CD) ratio was calculated.

### RT-qPCR analysis

The gene expression of jejunal mucosa was quantified using RT-qPCR, as described in our previous study [32]. Briefly, approximately 30 mg of each jejunal mucosa tissue was used for total RNA extraction with the SteadyPure Universal RNA Extraction Kit (AG21024, Accurate, Changsha, China). The extracted RNA was evaluated for quality and concentration using an ultramicro nucleic acid protein analyzer (BioDrop, Cambridge, UK). Subsequently, cDNA was synthesized with a reverse transcription kit (AG11728, Accurate, Changsha, China). The RT-qPCR was performed using a CFX Real-Time PCR Detection System (Bio-Rad, Hercules, CA, USA) with TransStart® Top Green qPCR SuperMix (GM0160, Accurate, Changsha, China). The primer information is provided in Table 2. The relative mRNA expression levels of the target genes were calculated using the 2^−ΔΔCt^ method [33], with β-actin serving as the internal control.

**Table 2:** Primer sequences utilized for RT-qPCR analysis

Gene	Accession number	Primer sequences (5′→ 3′)	Product size, bp
*IL-1β*	XM_021085847.1	F:CACACATGCTGAAGGCTCTC R:GGGTGGGCGTGTTATCTTTC	171
*IL-6*	NM_214399.1	F:CTGCAGTCACAGAACGAGTG R:GACGGCATCAATCTCAGGTG	131
*IL-10*	NM_214041.1	F:CTGAGAACAGCTGCATCCAC R:AAAGTCCTCCAGCAGAGACC	146
*TNF-α*	NM_214022.1	F:AAGGTCAACCTCCTCTCTGC R:CCTCCCAGGTAGATGGGTTC	98
*β-actin*	XM_003124280.5	F:CCCTGGAGAAGAGCTACGAG R:TAGAGGTCCTTGCGGATGTC	178

### Western blot analysis

The jejunal protein expression of tight junction proteins and key proteins related to the TLR4/NF-κB pathway was measured by Western blot, following the methodology of our previous study [34]. In brief, jejunal tissue proteins were separated by 10% SDS-PAGE and transferred onto a PVDF membrane using a trans-blotting system. The membrane was then blocked with 5% fat-free milk, and the proteins were subsequently detected using primary antibodies. The primary antibodies were as follows: occludin antibody (1:1,500; 27260-1-AP, Proteintech, Wuhan, China), ZO-1 antibody (1:1,500; 21773-1-AP, Proteintech, Wuhan, China), claudin-1 antibody (1:1,500; 28,674-1-AP, Proteintech, Wuhan, China), TLR4 antibody (1:1,500; 19811-1-AP, Proteintech, Wuhan, China), IκBα (1:1,000; 4812S, CST, Danvers, MA, USA), p-IκBα (1:1,500; 82349-1-RR, Proteintech, Wuhan, China), p65 (1:1,000; 8242S, CST, Danvers, MA, USA), p-p65 (1:1,000; 3033S, CST, Danvers, MA, USA) and β-actin antibody (1:1,500; 20536-1-AP, Proteintech, Wuhan, China). Bands were visualized and quantified using Bio-Rad ChemiDoc imaging systems (Bio-Rad, Hercules, CA, USA).

### Fecal microbiota analysis

Genomic DNA was extracted from fecal samples using the HiPure Stool DNA Kit (DC305-08, Alfa, Guangzhou, China), and its integrity was evaluated by 1% agarose gel electrophoresis. The purity and concentration of the extracted DNA were measured using a NanoDrop 2000 spectrophotometer (Thermo Fisher Scientific, Wilmington, USA). The V3–V4 region of bacterial 16S rRNA genes were amplified using the genomic DNA as a template, followed by purification of PCR products and sequencing on the Illumina HiSeq 2500 platform (Illumina, San Diego, USA). Data processing and bioinformatics analysis were conducted by Guangdong Magigene Biotechnology Co., Ltd. (Guangzhou, China). Briefly, raw sequences were processed utilizing the DADA2 pipeline (version 1.30.0) for quality filtration and denoising. Taxonomic classification was executed using the SILVA database (version 138.2). Bioinformatics analysis was performed on the QIIME 2 platform. α-Diversity indices (Shannon index, Chao1 index, and Simpson index) and β-diversity were computed. The relative abundance of taxa across treatment groups was analyzed to assess the effects of the experimental interventions. LEfSe analysis was also implemented to discern differentially abundant taxa.

### Statistical analysis

The statistical differences in β-diversity among treatment groups were assessed using permutational multivariate analysis of variance (PERMANOVA) in the QIIME 2 platform. A linear discriminant analysis (LDA) score threshold of 3 was applied in the LEfSe analysis to identify differentially abundant taxa in the QIIME 2 platform. Statistical analysis for other parameters was performed utilizing SPSS 24.0 software (SPSS Inc., Chicago, USA). Statistical differences among treatment groups were assessed using one-way analysis of variance (ANOVA) followed by Duncan’s multiple-range test. Spearman correlation analysis was performed to assess the correlation between altered gut microbiota and changes in diarrhea-related parameters, intestinal integrity, and inflammatory responses. A significance threshold of *P* < 0.05 was considered statistically significant, whereas 0.05 ≤ *P* < 0.10 was interpreted as a trend toward significance.

## Results

### Isolation, purification and transmission electron microscopy analysis of phages

**Fig. 1:**
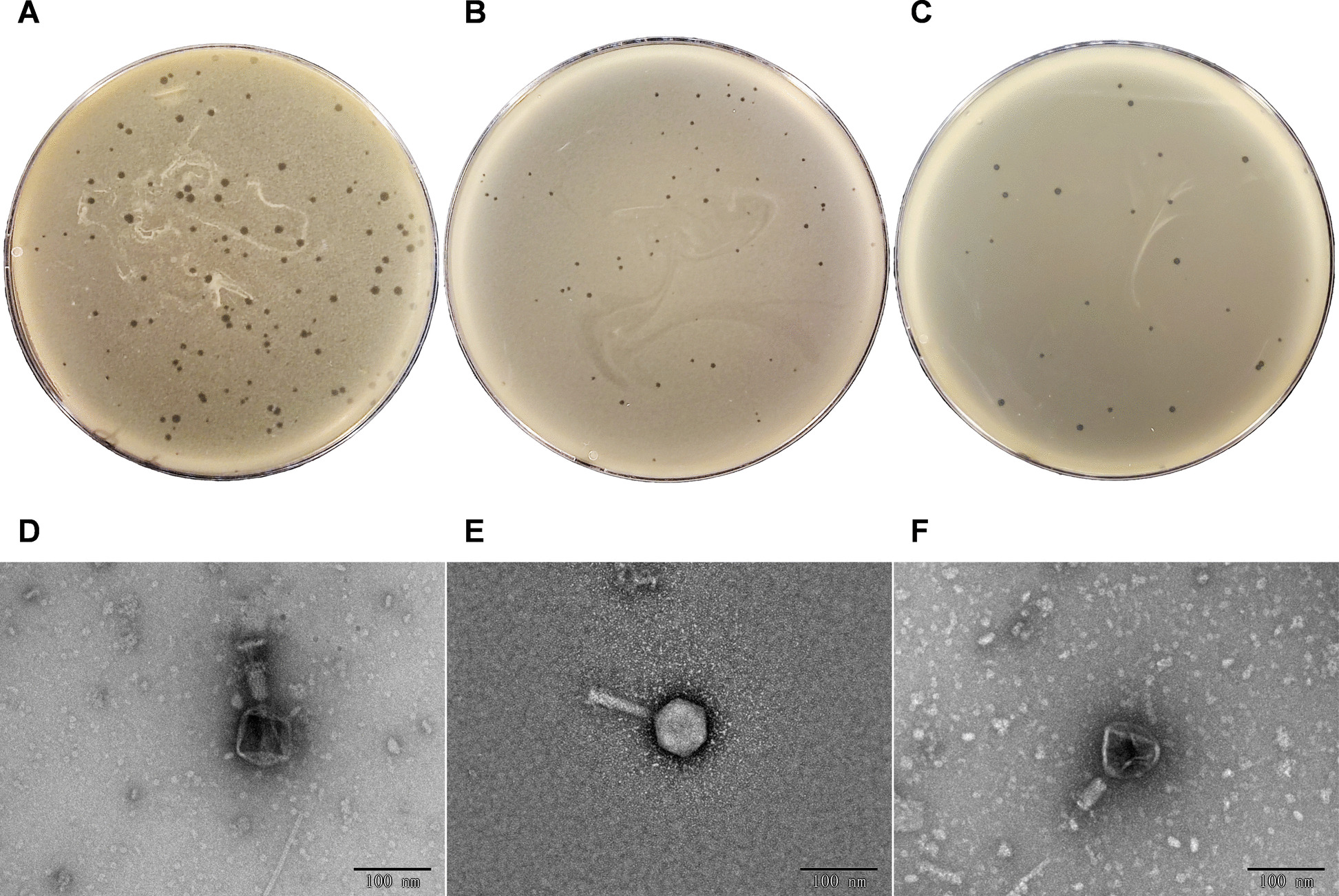
Plaques and transmission electron micrographs of the three isolated phages. **A**–**C** Plaques of phages EC-P1, EC-P2 and EC-P3. **D**–**F** Transmission electron micrographs of phages EC-P1, EC-P2 and EC-P3 (scale bar = 100 nm)

Three phages, designated as JXAU-EC-BP1 (EC-P1), JXAU-EC-BP2 (EC-P2), and JXAU-EC-BP3 (EC-P3), were isolated from pig farm sewage using the double-layer agar plate method with ETEC K88 (O141:K85, K88ab) strain as the host bacterium. Following purification, the phages formed small, clear plaques approximately 1 mm in diameter on the double-layer agar plates, characterized by translucent centers and distinct edges without a translucent halo (Fig. 1A–C). Transmission electron microscopy revealed that all three phages possessed typical tail structures (Fig. 1D–F). Phages EC-P1, EC-P2, and EC-P3 exhibited regular icosahedral heads with diameters of approximately 67 nm × 67 nm, 66 nm × 66 nm, and 76 nm × 76 nm, respectively, along with retractile tails measuring approximately 90 nm × 15 nm, 81 nm × 20 nm, and 85 nm × 25 nm, respectively.

### Genome information of isolated phages

**Fig. 2:**
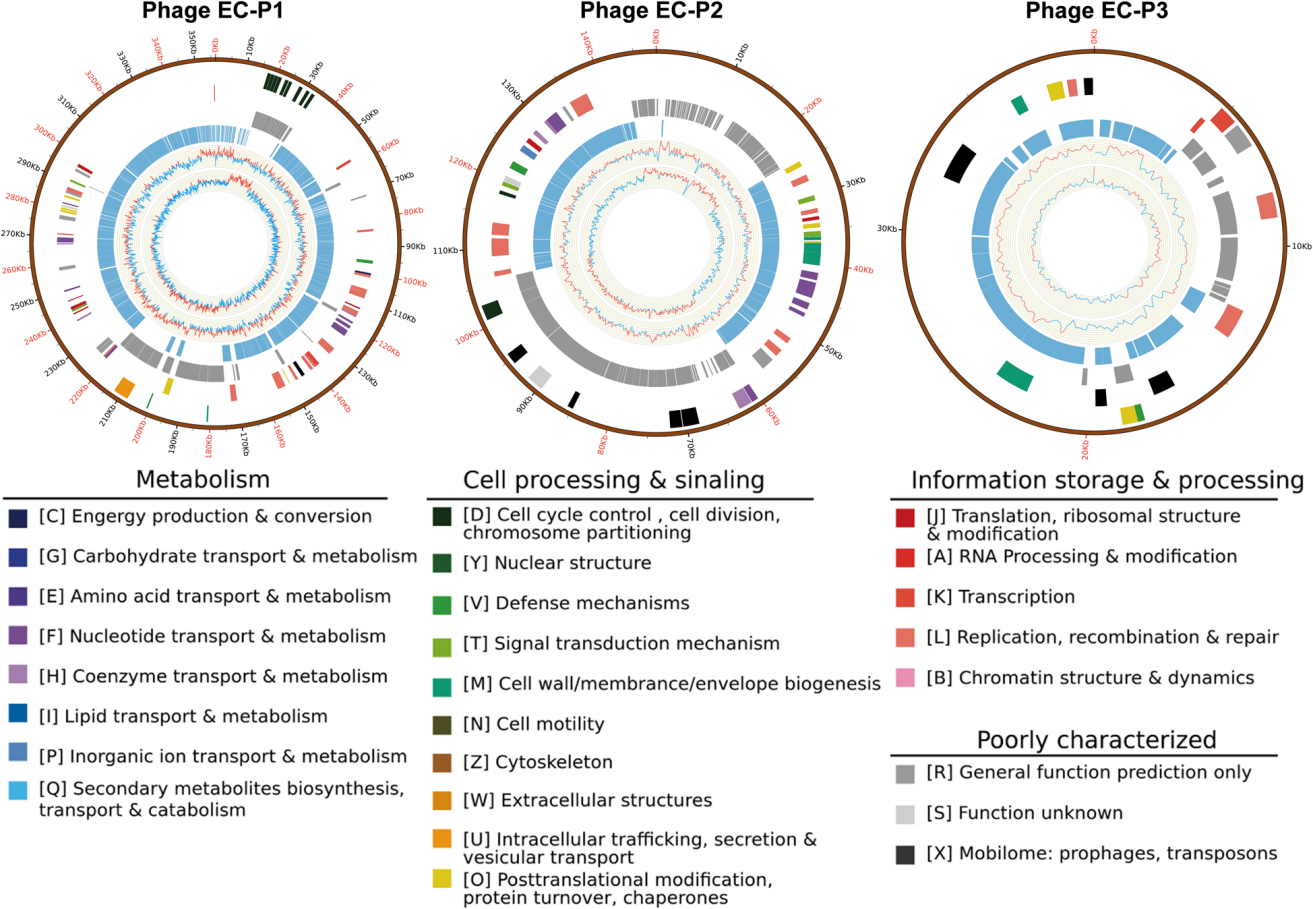
Schematic gene maps of the three isolated phages EC-P1, EC-P2, and EC-P3. Circles were arranged from inside to outside: G + C % content, GC skew plot, tRNA (dispensable), ORFs transcribed clockwise and counterclockwise denoted by specific colors according to their functional categories

The genome of phage EC-P1 is 327.57 kb in length, with a GC content of 35.69%. A total of 587 genes were predicted, of which 58 were functionally annotated in the UniProt database. Phage EC-P2 has a genome length of 133.72 kb and a GC content of 37.46%, with 241 predicted genes, 29 of which were functionally annotated in UniProt. Phage EC-P3, the smallest of the three, has a genome of 34.28 kb and a GC content of 47.20%, with 58 predicted genes, 36 of which were annotated in UniProt. By integrating target sequences with compositional analysis and functional annotation results, the viral genome data were visualized using Circos software. The resulting phage genome maps are presented in Fig. 2. The raw genome data of the three phages were deposited in the GenBank database of the National Center for Biotechnology Information (NCBI) under the following accession numbers: BankIt2899308 EC-P1 (PQ657772), BankIt2895556 EC-P2 (PQ631072), and BankIt2899079 EC-P3 (PQ657771).

### Biological characteristics of isolated phages

The lytic activity of three isolated phages (EC-P1, EC-P2, and EC-P3) against 13 common gut pathogenic bacteria is presented in Table 3. The results showed that phages EC-P1, EC-P2, and EC-P3 lysed 43.75%, 50.00%, and 75.00% of the tested strains, respectively. In addition to *E. coli*, EC-P3 also lysed two *Salmonella typhimurium* strains (CICC 21483 and CICC 21484). However, none of the three phages exhibited lytic activity against the *Staphylococcus aureus* strain (CICC 21600).

**Table 3:** The host range of the three isolated phages

No.	Species	Strain	Lysis activity		
			EC-P1	EC-P2	EC-P3
1	Enterotoxigenic *Escherichia coli*	CVCC 195	−	+	+
2	Enterotoxigenic *Escherichia coli*	CVCC 199	+	−	+
3	Enterotoxigenic *Escherichia coli*	CVCC 200	+	−	+
4	Enterotoxigenic *Escherichia coli*	CICC 10413	+	+	+
5	Enterotoxigenic *Escherichia coli*	CICC 10414	−	−	+
6	Enterotoxigenic *Escherichia coli*	CICC 10415	+	+	−
7	Enteropathogenic *Escherichia coli*	CICC 10372	+	+	+
8	Enteropathogenic *Escherichia coli*	CICC 10411	−	+	+
9	Enteropathogenic *Escherichia coli*	CICC 10412	+	+	−
10	Enterohemorrhagic *Escherichia coli*	CICC 21530	+	+	+
11	*Salmonella typhimurium*	CICC 21483	−	−	+
12	*Salmonella typhimurium*	CICC 21484	−	−	+
13	*Staphylococcus aureus*	CICC 21600	−	−	−

+ = lytic; – = non-lytic

**Fig. 3:**
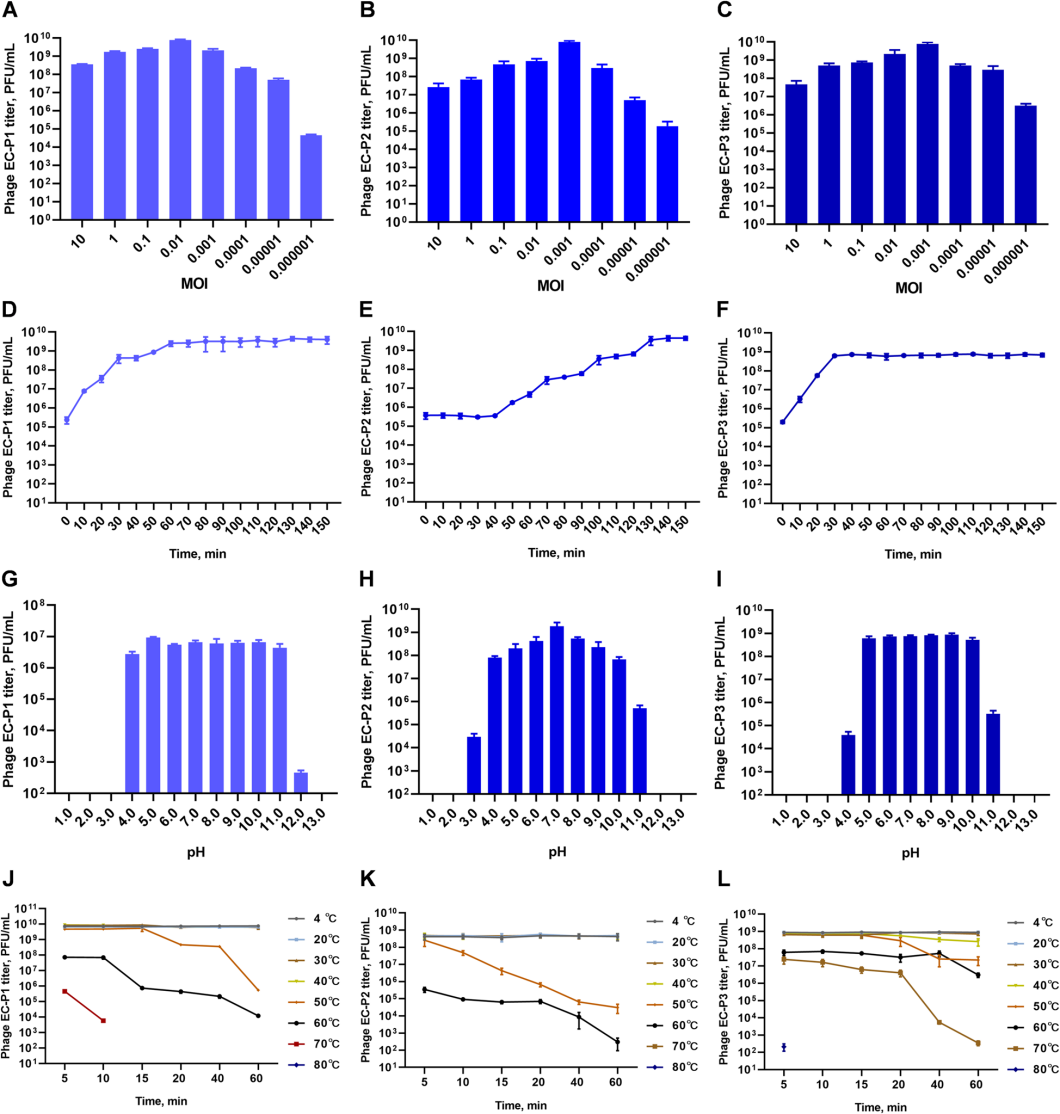
Biological characteristics of the three isolated phages (mean ± SEM; *n* = 3). **A**–**C** Optimal multiplicity of infection (MOI) of phages EC-P1, EC-P2, and EC-P3. **D**–**F** One-step growth curves of phages EC-P1, EC-P2, and EC-P3. **G**–**I** Acid–base stability of phages EC-P1, EC-P2, and EC-P3. **J**–**L** Thermal stability of phages EC-P1, EC-P2, and EC-P3

The optimal MOI for the three isolated phages is shown in Fig. 3A–C. The titer of phage EC-P1 was highest at 7.84 × 10^9^ PFU/mL with an MOI of 0.01. Additionally, at an MOI of 0.001, the titers of phages EC-P2 and EC-P3 peaked at 8.01 × 10^9^ PFU/mL and 7.70 × 10^9^ PFU/mL, respectively.

The one-step growth curves of the phages are shown in Fig. 3D–F. Phages EC-P1 and EC-P3 exhibited no latency period and proliferated rapidly from 0 to 30 min, reaching titers of 2.56 × 10^9^ PFU/mL and 6.29 × 10^8^ PFU/mL, respectively, before stabilizing. In contrast, phage EC-P2 displayed a 40-min latency period, followed by a steady increase in titer to 3.60 × 10^9^ PFU/mL, after which it remained stable.

The acid–base stability of the phages is illustrated in Fig. 3G–I. Phage EC-P1 retained its activity within a pH range of 4.0–11.0 but was completely inactive below pH 4.0 or above pH 11.0. Phage EC-P2 remained stable within a pH range of 3.0–11.0; however, its titer decreased by 2.92 × 10^4^ PFU/mL at pH 3.0 and 5.1 × 10^5^ PFU/mL at pH 11.0. Phage EC-P3 maintained stability within a pH range of 4.0–11.0 but was inactive outside this range.

The thermal stability of the phages is shown in Fig. 3J–L. Phages EC-P1 and EC-P2 exhibited stability at temperatures ranging from 4 to 40 °C for 1 h but demonstrated sensitivity to elevated temperatures. The titer of phage EC-P1 declined to 5.83 × 10^3^ PFU/mL after exposure to 70 °C for 10 min and was completely inactivated within 20 min. Phage EC-P2 exhibited a gradual reduction in titer at 50–60 °C and rapid inactivation at temperatures over 70 °C. EC-P3 remained stable at 4–60 °C for 1 h; however, its titer decreased at temperatures exceeding 70 °C and was fully inactivated after 5 min at 80 °C.

### The in vitro inhibitory effects of isolated phages on ETEC K88

**Fig. 4:**
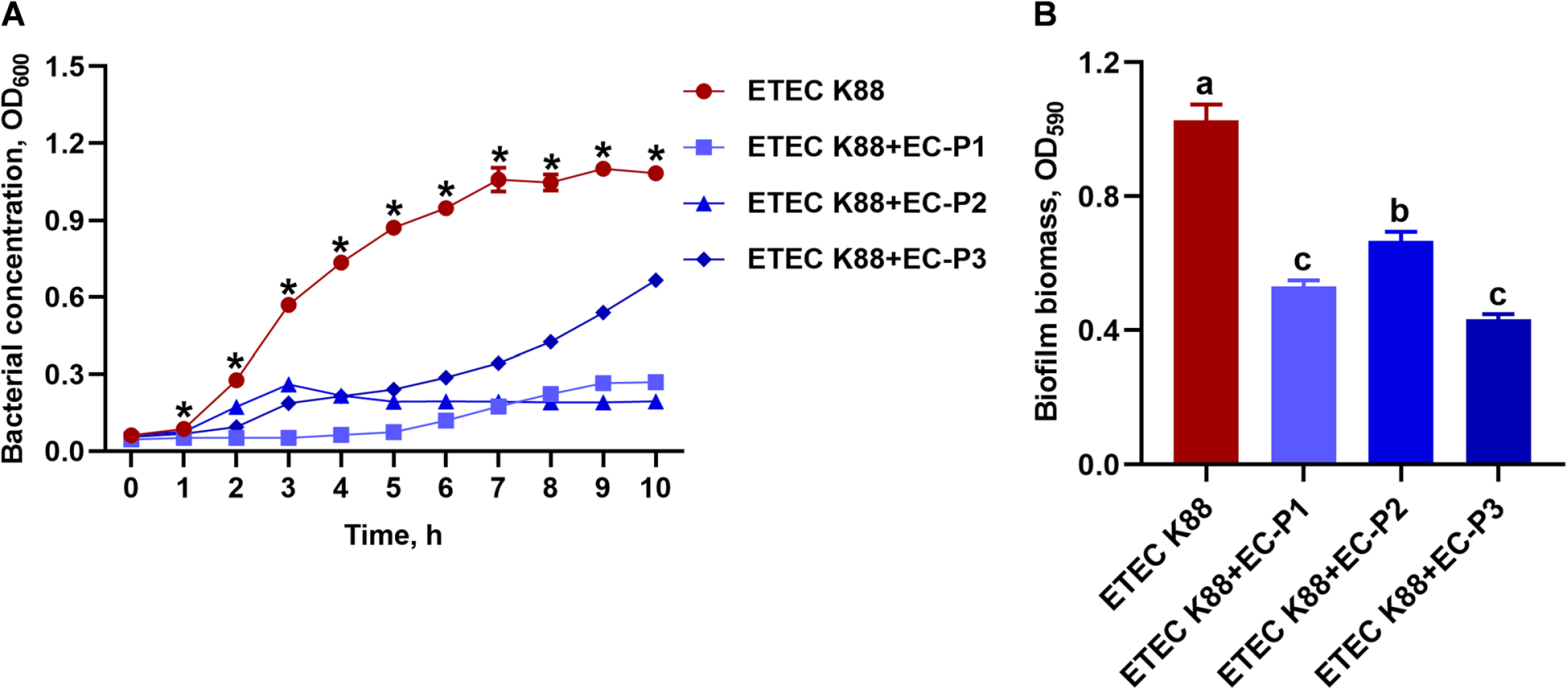
The in vitro inhibitory effects of the three isolated phages on ETEC K88 (mean ± SEM; *n* = 3). **A** Antibacterial activity of isolated phages against ETEC K88. ^*^P < 0.05 for the *E. coli* group compared to all ETEC K88 + phages groups (EC-P1, EC-P2, and EC-P3). **B** Inhibitory activity of isolated phages on biofilm formation of ETEC K88. Different superscript letters denote statistically significant differences among treatment groups (*P* < 0.05)

The antibacterial activity of isolated phages against ETEC K88 is presented in Fig. 4A. The ETEC K88 concentration was significantly decreased after 1–10 h of treatment with phages EC-P1, EC-P2, or EC-P3 (*P* < 0.05). Additionally, the ETEC K88 biofilm was markedly reduced by 48.35%, 35.13%, and 57.92% following treatment with phages EC-P1, EC-P2, and EC-P3, respectively (*P* < 0.05) (Fig. 4B).

### Effects of dietary supplementation with lyophilized powder of isolated phages on growth performance and diarrhea characteristics

As shown in Table 4, the growth performance of piglets, including final body weight, ADG, ADFI, and FCR, was not statistically affected by the experimental treatments (*P* > 0.05). Compared with the CON group, the diarrhea scores and rectal temperatures of piglets in the ETEC K88 group were elevated at 12, 24, and 48 h post-infection (*P* < 0.05). However, compared with the ETEC K88 group, diarrhea scores of piglets were decreased at 12 and 24 h post-infection, while rectal temperatures of piglets were reduced at 24 and 48 h post-infection in the ETEC K88 + 400 mg/kg phages group (*P* < 0.05). More importantly, compared to the ETEC K88 group, the diarrhea scores and rectal temperatures of piglets in the ETEC K88 + 600 mg/kg phages group and ETEC K88 + 800 mg/kg phages group were significantly lower at 12, 24, and 48 h post-infection (*P* < 0.05). It should be noted that diarrhea scores and rectal temperatures did not significantly differ between the ETEC K88 + 800 mg/kg phages group and the ETEC K88 + 600 mg/kg phages group (*P* > 0.05). Therefore, the CON group, ETEC K88 group, and ETEC K88 + 600 mg/kg phages group were selected for the subsequent study.

**Table 4:** Effects of isolated phages on growth performance and diarrhea characteristics of weaned piglets infected with ETEC K88 (*n* = 6)

Item	CON	ETEC K88	ETEC K88 + 400 mg/kg phages	ETEC K88 + 600 mg/kg phages	ETEC K88 + 800 mg/kg phages	SEM	*P-*value
Initial body weight, kg	6.33	6.48	6.38	6.53	6.53	0.09	0.893
Final body weight, kg	9.27	9.38	9.13	9.55	9.60	0.15	0.814
Average daily gain, g	209.5	196.4	207.1	215.5	219.1	6.0	0.818
Average daily feed intake, g	383.2	399.5	397.1	418.1	424.7	6.5	0.526
Feed conversion ratio	1.87	2.04	1.99	1.96	1.96	0.06	0.929
Diarrhea score							
12 h post-infection	1.17^c^	3.50^a^	2.00^b^	1.83^bc^	1.67^bc^	0.26	0.001
24 h post-infection	1.50^b^	2.50^a^	1.50^b^	1.33^b^	1.17^b^	0.20	< 0.001
48 h post-infection	1.00^b^	1.67^a^	1.33^ab^	1.00^b^	1.00^b^	0.13	0.030
Rectal temperature, °C							
12 h post-infection	38.88^c^	40.86^a^	40.22^ab^	39.65^bc^	39.50^bc^	0.19	0.020
24 h post-infection	38.87^c^	41.58^a^	39.95^b^	39.05^c^	38.97^c^	0.25	< 0.001
48 h post-infection	38.73^b^	40.56^a^	38.96^b^	38.62^b^	38.65^b^	0.19	< 0.001

Different superscript letters denote statistically significant differences among treatment groups (*P* < 0.05)

### Effects of dietary supplementation with lyophilized powder of isolated phages on fecal dry matter content and ETEC K88 load

**Fig. 5:**
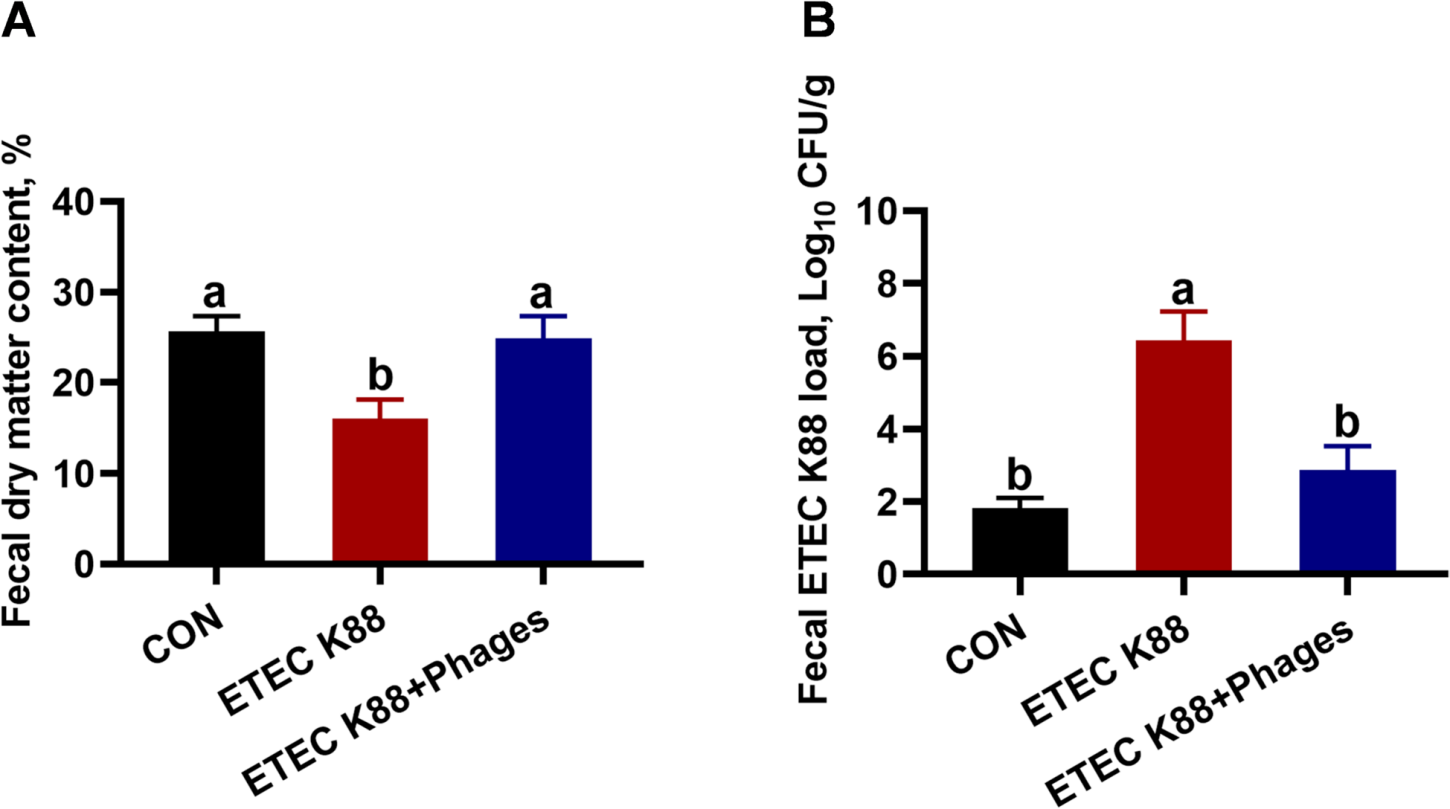
Fecal dry matter content and ETEC K88 load in piglets at 48 h post-infection in response to the three treatments (mean ± SEM; *n* = 6). **A** Fecal dry mater content. **B** Fecal ETEC K88 load. Different superscript letters denote statistically significant differences among treatment groups (*P* < 0.05)

As shown in Fig. 5, ETEC K88 infection reduced fecal dry matter content and increased fecal ETEC K88 load in piglets at 48 h post-infection compared to the CON group (*P* < 0.05). In contrast, piglets in the ETEC + phages group exhibited higher fecal dry matter content and lower fecal ETEC K88 load at 48 h post-infection compared with the ETEC K88 group (*P* < 0.05).

### Effects of dietary supplementation with lyophilized powder of isolated phages on intestinal integrity

**Fig. 6:**
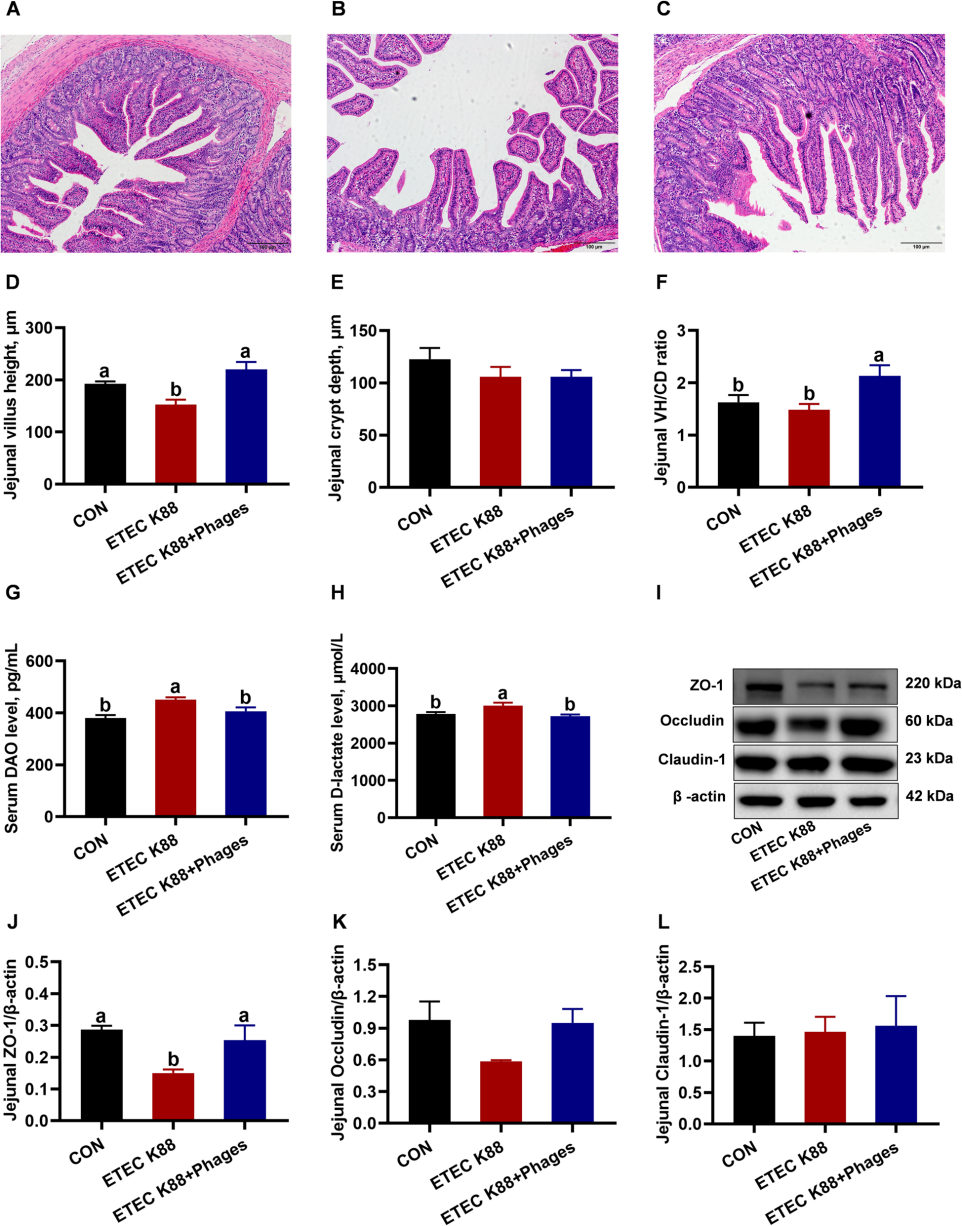
Intestinal integrity of piglets in response to the three treatments (mean ± SEM; *n* = 6, except *n* = 3 for Western blot analysis). **A**–**C** H&E images of jejunal morphology in the CON, ETEC K88, and ETEC K88 + phages groups (scale bar = 100 μm). **D**–**F** Jejunal villus height, crypt depth, and villus height-to-crypt depth (VH/CD) ratio. **G** and **H** Serum diamine oxidase (DAO) and D-lactate levels. **I** Representative Western blot bands. **J**–**L** Jejunal ZO-1, Occludin, and Claudin-1 protein expression levels. Different superscript letters denote statistically significant differences among treatment groups (*P* < 0.05)

As displayed in Fig. 6, compared with the CON group, the jejunal villus height of piglets in the ETEC K88 group was decreased, while the jejunal villus height-to-crypt depth ratio was increased in the ETEC K88 + phages (600 mg/kg) group relative to the ETEC K88 group (*P* < 0.05). Additionally, serum DAO and D-lactate levels were higher in the ETEC K88 group than in the CON group but were reduced in the ETEC K88 + phages group compared to the ETEC K88 group (*P* < 0.05). Furthermore, jejunal ZO-1 protein expression was downregulated in the ETEC K88 group versus the CON group but upregulated in the ETEC K88 + phages group relative to the ETEC K88 group (*P* < 0.05).

### Effects of dietary supplementation with lyophilized powder of isolated phages on inflammatory responses

**Fig. 7:**
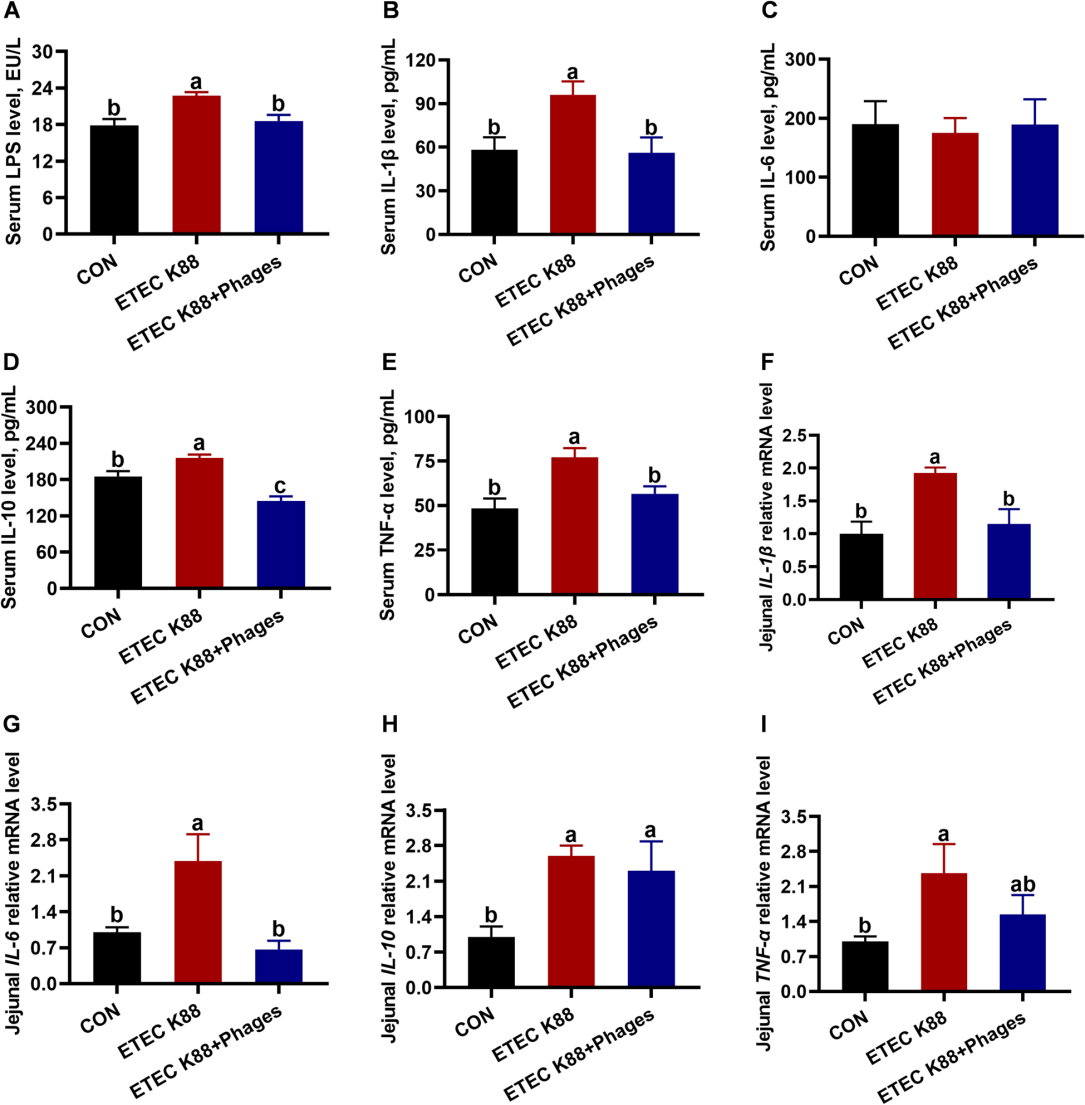
Systemic and jejunal inflammation of piglets in response to the three treatments (mean ± SEM; *n* = 6). **A**–**E** Serum LPS, IL-1β, IL-6, IL-10, and TNF-α levels. **F**–**H** Jejunal relative mRNA expression levels of *IL-1β*, *IL-6*, *IL-10*, and *TNF-α*. Different superscript letters denote statistically significant differences among treatment groups (*P* < 0.05)

As illustrated in Fig. 7, serum levels of LPS, IL-1β, IL-10, and TNF-α in piglets were elevated in the ETEC K88 group compared to the CON group, whereas these levels were reduced in the ETEC K88 + phages group relative to the ETEC K88 group (*P* < 0.05). Compared with the CON group, the relative mRNA levels of *IL-1β*, *IL-6*, *IL-10*, and *TNF-α* in the jejunum of piglets were upregulated in the ETEC K88 group. However, the relative mRNA levels of *IL-1β* and *IL-6* were downregulated in the ETEC K88 + phages group versus the ETEC K88 group (*P* < 0.05).

**Fig. 8:**
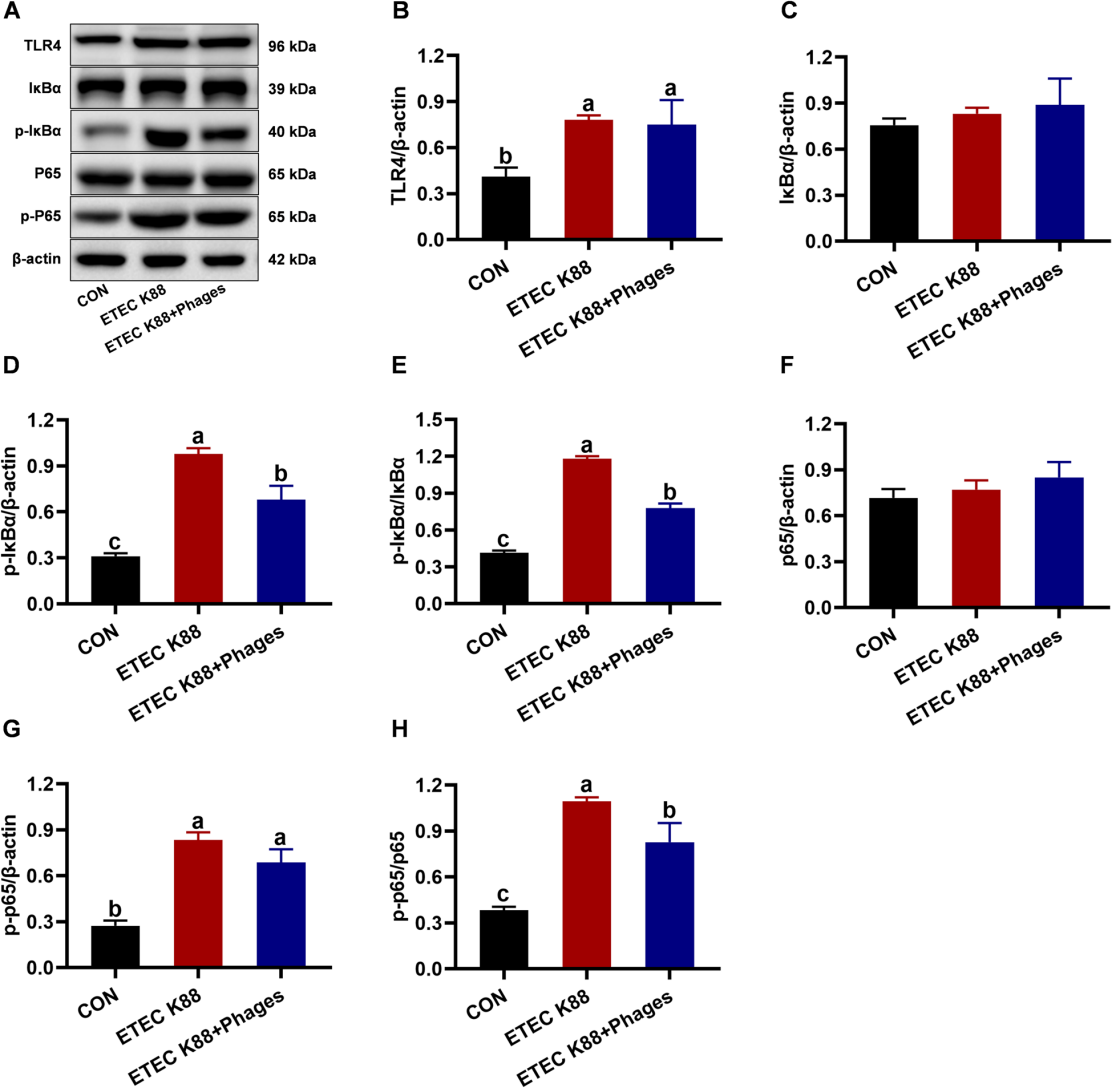
Jejunnal protein expression related to TLR4/NF-κB signaling pathway in piglets in response to the three treatments (mean ± SEM; *n* = 3). **A** Representative Western blot bands. **B**–**H** Jejunal protein expression levels of TLR-4, IκBα, p-IκBα, p-IκBα/IκBα, p65, p-p65, and p-p65/p65. Different superscript letters denote statistically significant differences among treatment groups (*P* < 0.05)

The jejunal protein expression related to TLR4/NF-κB signaling pathway in piglets in response to the three treatments is shown in Fig. 8. Compared with the CON group, the protein expression levels of TLR4, p-IκBα, p-IκBα/IκBα, p-p65, and p-p65/p65 in the jejunum of piglets were significantly upregulated in the ETEC K88 group (*P* < 0.05). In contrast, the protein expression levels of p-IκBα, p-IκBα/IκBα, and p-p65/p65 were markedly downregulated in the ETEC K88 + phages group compared to the ETEC K88 group (*P* < 0.05).

### Effects of dietary supplementation with lyophilized powder of isolated phages on fecal short-chain fatty acid levels

**Fig. 9:**
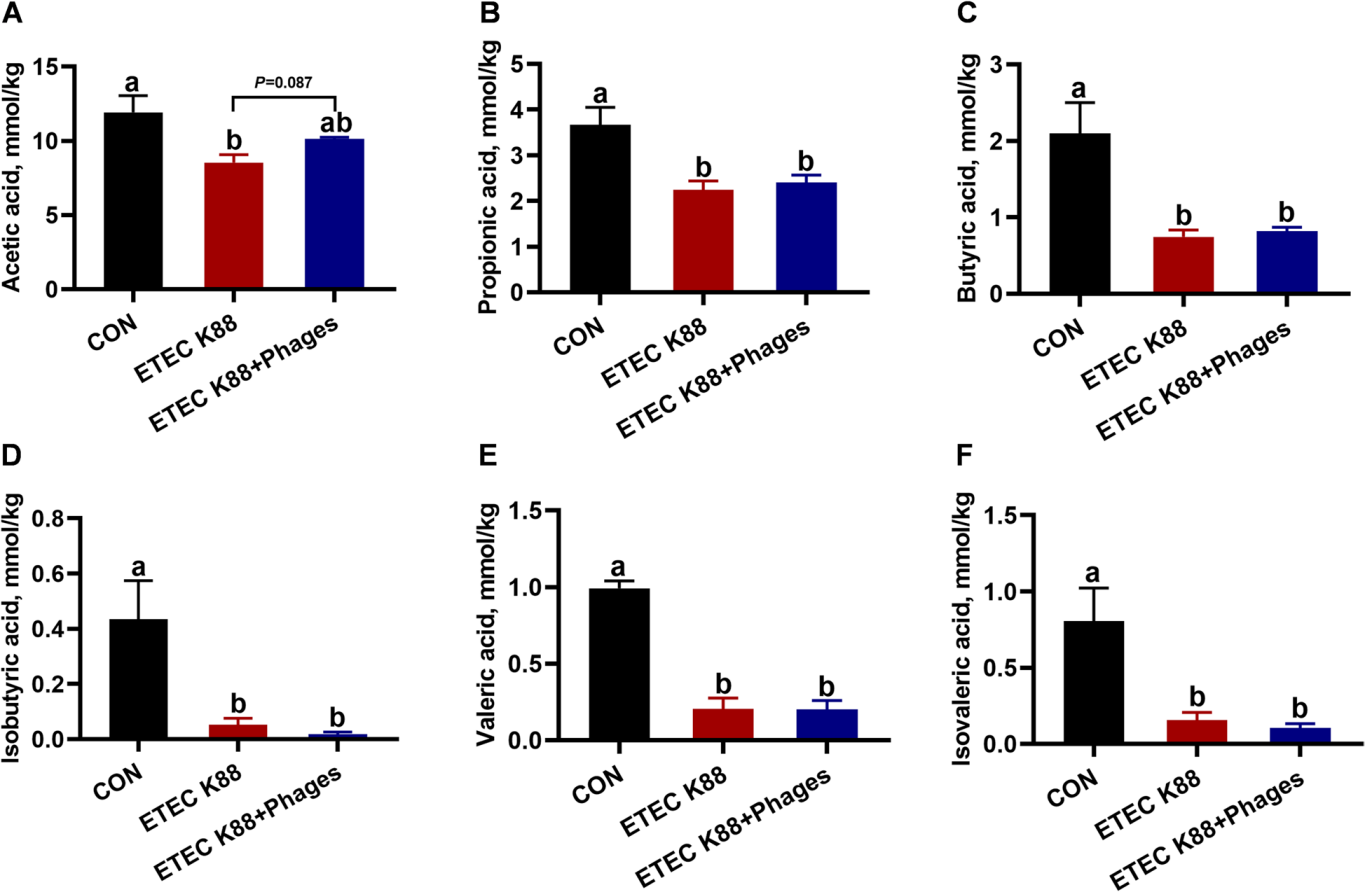
Fecal short-chain fatty acid levels of piglets across the three experimental treatments (mean ± SEM; *n* = 6). **A** Acetic acid. **B** Propionic acid. **C** Butyric acid. **D** Isobutyric acid. **E** Valeric acid. **F** Isovaleric acid. Different superscript letters denote statistically significant differences among treatment groups (*P* < 0.05)

As shown in Fig. 9, compared with the CON group, ETEC K88 infection significantly reduced the concentrations of acetic acid, propionic acid, butyric acid, isobutyric acid, and valeric acid in the feces of piglets (*P* < 0.05). However, dietary supplementation with 400 mg/kg phages tended to increase the fecal acetic acid concentration in piglets compared to the ETEC K88 group (*P* = 0.087).

### Effects of dietary supplementation with lyophilized powder of isolated phages on diversity of gut microbiota

**Fig. 10:**
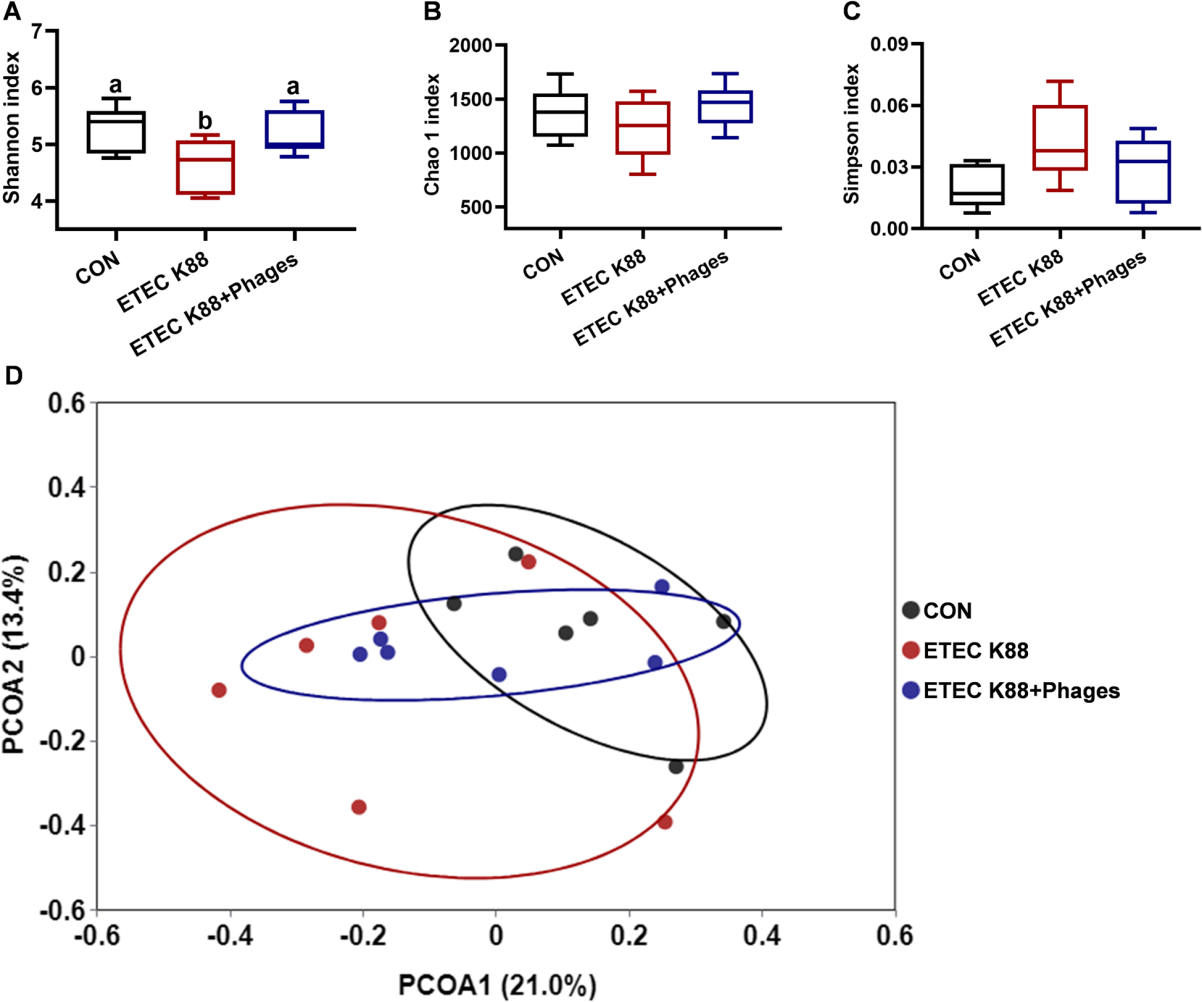
Fecal microbiota diversity in piglets across the three experimental treatments (*n* = 6). **A** Shannon index. **B** Chao 1 index. **C** Simpson index. **D** β-diversity (Adonis analysis: *P* = 0.034 for ETEC K88 group vs. CON group; *P* = 0.096 for ETEC K88 + phages group vs. ETEC K88 group; *P* = 0.047 for ETEC K88 + phages group vs. CON group). Different superscript letters denote statistically significant differences among treatment groups (*P* < 0.05)

As shown in Fig. 10, the Chao 1 index and Simpson index of fecal microbiota were unaffected by experimental treatments (*P* > 0.05). However, compared to the CON group, the Shannon index was decreased in the ETEC K88 group, while this index was increased in the ETEC K88 + phages group relative to the ETEC K88 group (*P* < 0.05). Regarding β-diversity, a significant difference was observed between the ETEC K88 group and the CON group (*P* = 0.034), with a trend toward difference between the ETEC K88 + phages group and the ETEC K88 group (*P* = 0.096).

### Effects of dietary supplementation with lyophilized powder of isolated phages on composition of gut microbiota

**Fig. 11:**
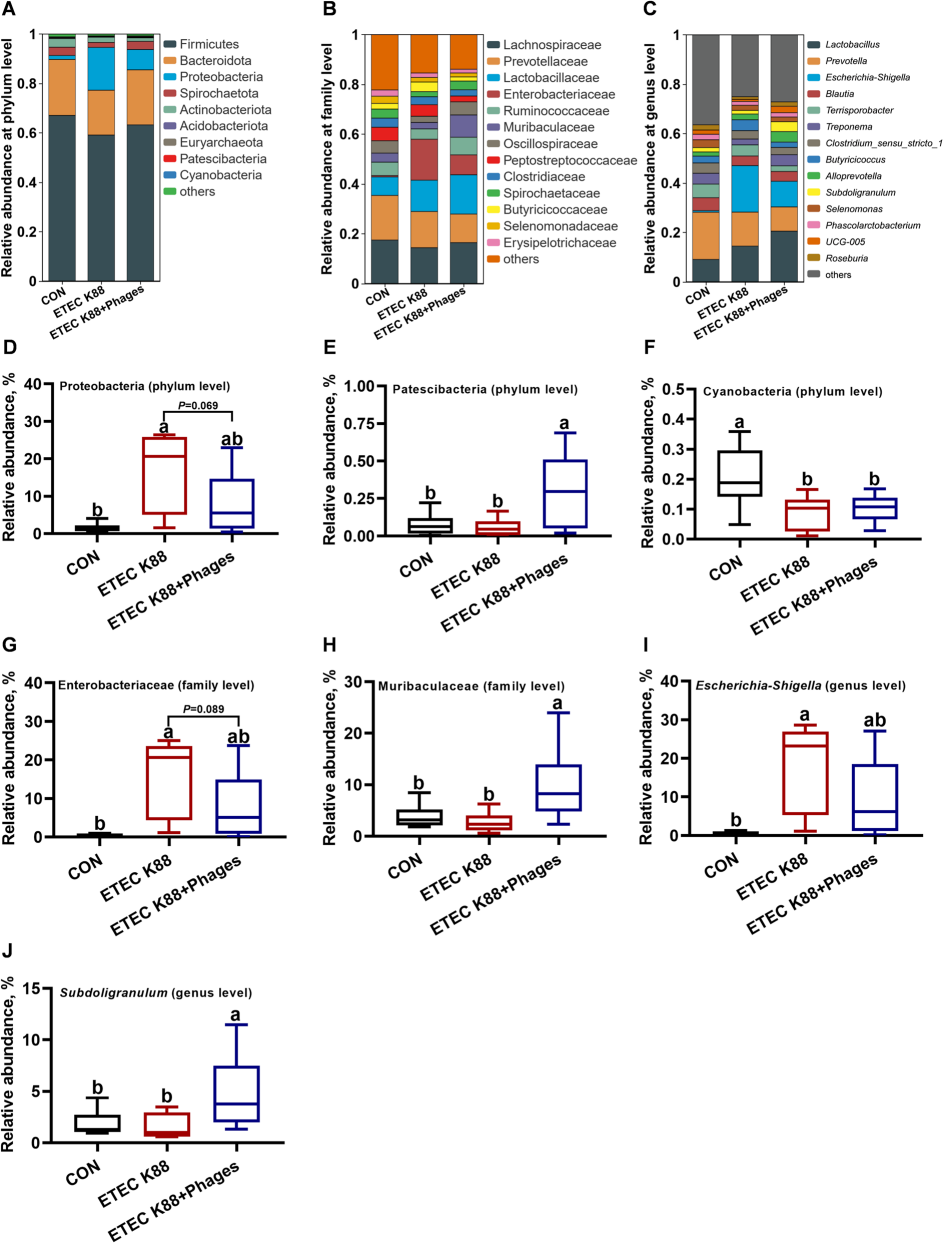
Microbial composition at the phylum, family, and genus levels in the feces of piglets across the three experimental treatments (*n* = 6). **A**–**C** Relative abundance at the phylum, family, and genus levels. **D**–**J** Relative abundance of Proteobacteria, Patescibacteria, Cyanobacteria, Enterobacteriaceae, Muribaculaceae, *Escherichia-Shigella*, and *Subdoligranulum*. Different superscript letters denote statistically significant differences among treatment groups (*P* < 0.05)

Figure 11 illustrates the microbial composition at the phylum, family, and genus levels in the feces of piglets across the three experimental treatments. Compared to the CON group, the relative abundance of Proteobacteria, Enterobacteriaceae, and *Escherichia-Shigella* was increased, while the relative abundance of Cyanobacteria was decreased in the ETEC K88 group (*P* < 0.05). However, the relative abundance of Proteobacteria and Enterobacteriaceae showed a tendency to decrease in the ETEC K88 + phages group compared to the ETEC K88 group (*P* < 0.10). Additionally, compared to the ETEC K88 group, the relative abundance of Patescibacteria, Muribaculaceae and *Subdoligranulum* was increased in the ETEC K88 + phages group (*P* < 0.05).

**Fig. 12:**
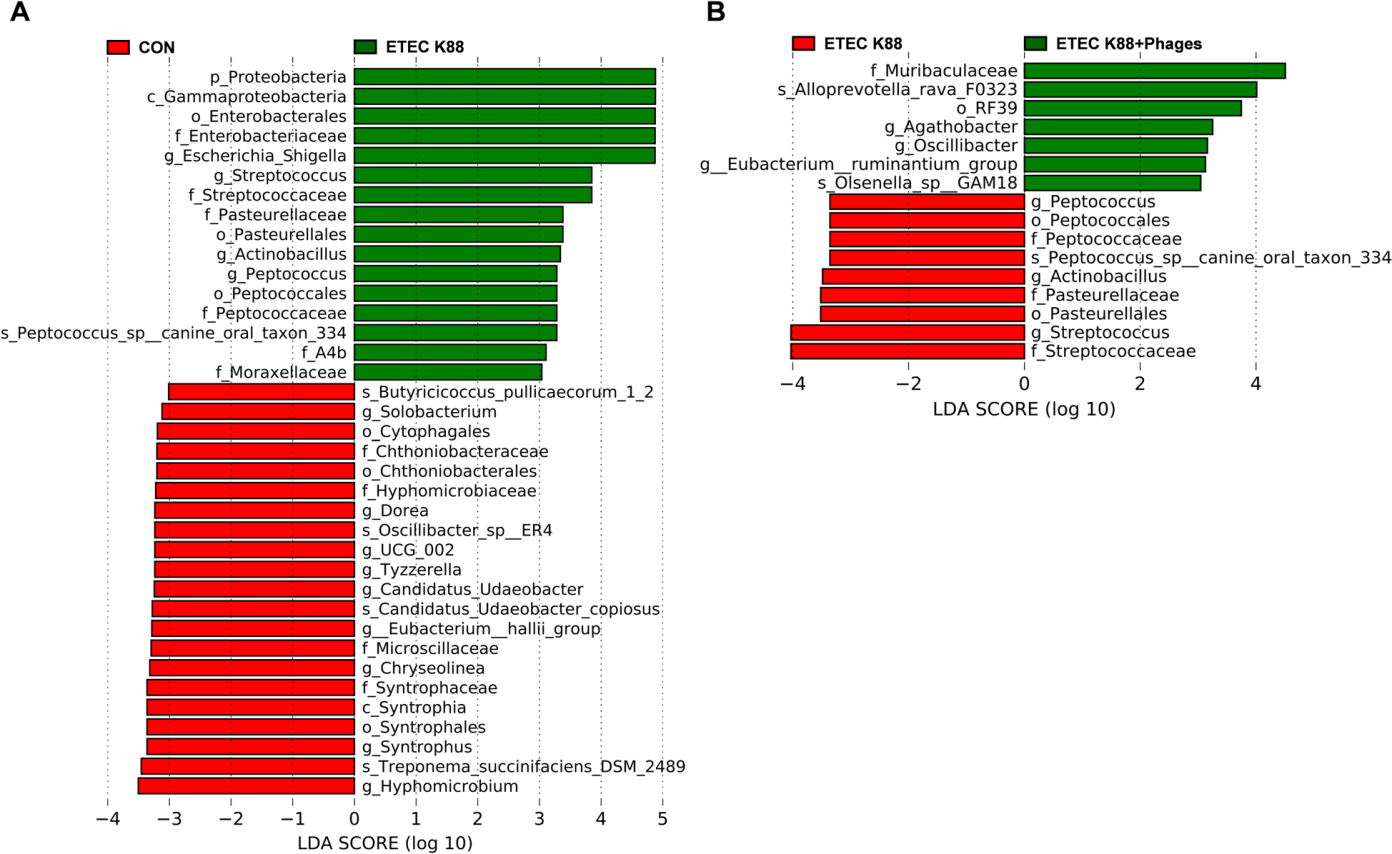
LEfSe analysis of the fecal microbial community in piglets (LDA scores > 3.0; *n* = 6). **A** LEfSe analysis comparing the ETEC K88 and CON groups. **B** LEfSe analysis comparing the ETEC K88 + phages and ETEC K88 groups

LEfSe analysis was subsequently performed to explore specific alterations in fecal microbiota at the phylum (p), class (c), order (o), family (f), genus (g), and species (s) levels among piglets in response to the experimental treatments (Fig. 12). Compared with the CON group, ETEC K88 infection increased abundances of Proteobacteria (p), Gammaproteobacteria (c), Enterobacterales (o), Enterobacteriaceae (f), *Escherichia-Shigella* (g), *Streptococcus* (g), Streptococcaceae (f), Pasteurellaceae (f), Pasteurellales (o), *Actinobacillus* (g), *Peptococcus* (g), Peptococcales (o), Peptococcaceae (f), *Peptococcus_sp__canine_oral_taxon_334* (s), A4b (f), and Moraxellaceae (f). Conversely, the abundances of following microbiota were decreased: *Butyricicoccus_pullicaecorum_1_2* (s), *Solobacterium* (g), Cytophagales (o), Chthoniobacteraceae (f), Chthoniobacterales (o), Hyphomicrobiaceae (f), *Dorea* (g), *Oscillibacter_sp__ER4* (s), *UCG_002* (g), *Tyzzerella* (g), *Candidatus_Udaeobacter* (g), *Candidatus_Udaeobacter_copiosus* (s), *Eubacterium__hallii_group* (g), Microscillaceae (f), *Chryseolinea* (g), Syntrophaceae (f), Syntrophia (c), Syntrophales (o), *Syntrophus* (g), *Treponema_succinifaciens_DSM_2489* (s), and *Hyphomicrobium* (g). Compared with the ETEC K88 group, ETEC K88 + phages group exhibited increased abundances of Muribaculaceae (f), *Alloprevotella_rava_F0323* (s), RF39 (o), *Agathobacter* (g), *Oscillibacter* (g), *Eubacterium__ruminantium_group* (g), and *Olsenella_sp__GAM18* (s). In contrast, the abundances of *Peptococcus* (g), Peptococcales (o), Peptococcaceae (f), *Peptococcus_sp__canine_oral_taxon_334* (s), *Actinobacillus* (g), Pasteurellaceae (f), Pasteurellales (o), *Streptococcus* (g), and Streptococcaceae (f) were decreased in the ETEC K88 + phages group vs. ETEC K88 group.

### Correlation analysis between gut microbiota and diarrhea characteristics, intestinal integrity, and inflammatory responses

**Fig. 13:**
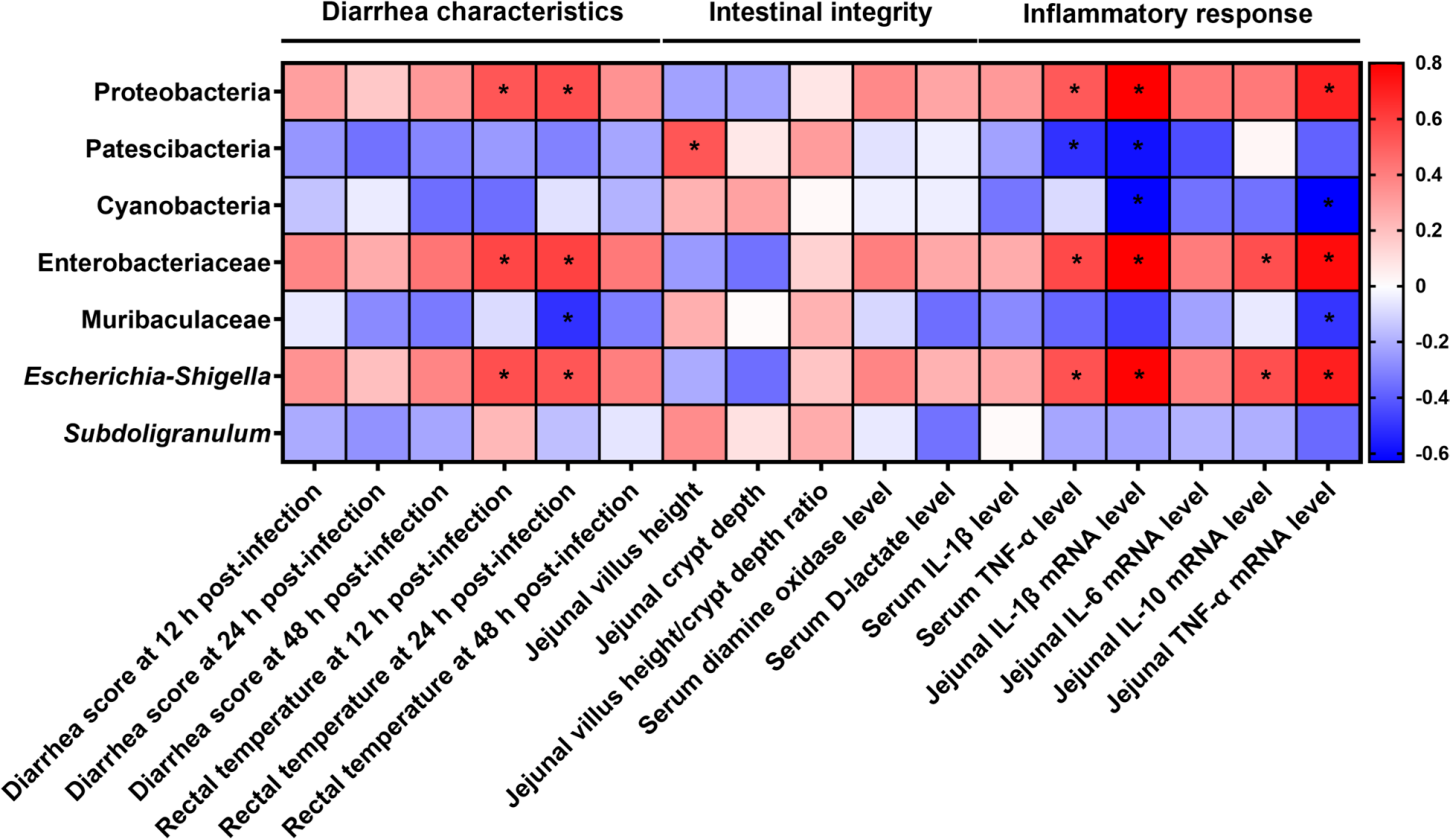
Spearman correlation analysis between altered gut microbiota and changes in parameters related to diarrhea characteristics, intestinal integrity, and inflammatory responses. Significant correlations are indicated by ^*^P < 0.05

Figure 13 displays the Spearman correlation analysis between altered gut microbiota and changes in parameters related to diarrhea characteristics, intestinal integrity, and inflammatory responses. The abundance of Proteobacteria exhibited a positive correlation with rectal temperature at both 12 and 24 h post-infection, as well as with serum TNF-α levels and jejunal *IL-1β* and *TNF-α* mRNA levels. Moreover, the abundance of Patescibacteria was positively correlated with jejunal villus height but negatively correlated with serum TNF-α level and jejunal *IL-1β* mRNA level. Cyanobacteria abundance, on the other hand, displayed a negative correlation with jejunal *IL-1β* and *TNF-α* mRNA levels. The abundance of Enterobacteriaceae or *Escherichia-Shigella* was positively correlated with rectal temperature at 12 and 24 h post-infection, serum TNF-α level, and jejunal *IL-1β*, *IL-10*, and *TNF-α* mRNA levels. Besides, the abundance of Muribaculaceae was negatively correlated with rectal temperature at 24 h post-infection and jejunal *TNF-α* mRNA level.

## Discussion

Since ETEC K88 is the primary causative pathogen of PWD in piglets [3, 4], this study aimed to isolate phages specific to ETEC K88 using the strain as the host bacterium. Additionally, the efficacy of dietary phage supplementation was evaluated in newly-weaned piglets over a two-week period using an ETEC K88 challenge model. In this study, three lytic ETEC K88-specific phages (EC-P1, EC-P2, and EC-P3), belonging to the tailed phage group, were isolated. The MOI values of these phages remained relatively low, effectively reducing application costs in practical production and enabling large-scale phage manufacturing. Besides, these phages exhibit a short latent period and a large burst size, allowing rapid host attachment and efficient proliferation, which could promote the lysis of pathogenic bacteria. The isolated phages demonstrated high activity across a broad pH range (3.0–11.0), indicating compatibility with the pH conditions of the piglet gastrointestinal tract [35]. In vitro bacteriostatic assays demonstrated significant efficacy in reducing ETEC K88 loads and inhibiting ETEC K88 biofilm formation, indicating potential applications for weaned piglets in vivo. Therefore, we prepared lyophilized isolated phage powder and performed a pilot study to evaluate the efficacy of dietary phage supplementation in weaned piglets over a two-week period using an ETEC K88 challenge model.

In the ETEC K88 challenge study with weaned piglets, we observed that ETEC K88 infection resulted in increased diarrhea scores at 12, 24, and 48 h post-infection, accompanied by elevated rectal temperatures at the same time points. Besides, ETEC K88 infection decreased fecal dry matter content and elevated fecal ETEC K88 load in piglets at 48 h post-infection compared with the CON group. These diarrhea characteristics indicate the successful establishment of ETEC K88-induced diarrhea [36]. Interestingly, dietary supplementation with 400, 600, or 800 mg/kg lyophilized isolated phage powder reduced diarrhea scores and rectal temperatures in piglets infected with ETEC K88. The additive effects of phages were further supported by the higher fecal dry matter content and lower fecal ETEC K88 load observed in piglets from the ETEC + phages group compared to the ETEC K88 group at 48 h post-infection. However, no significant differences were observed in diarrhea scores or rectal temperatures between the ETEC K88 + 800 mg/kg phages group and the ETEC K88 + 600 mg/kg phages group. Consequently, the CON group, ETEC K88 group, and ETEC K88 + 600 mg/kg phages group were selected for further analysis.

The intestinal systems of weaned piglets remain underdeveloped [37]. ETEC K88 colonizes intestinal epithelium through fimbrial adhesion and compromises intestinal integrity [38, 39]. Compared with the CON group, the jejunal villus height of piglets in the ETEC K88 group was decreased, while the jejunal villus height-to-crypt depth ratio was increased in the ETEC K88 + phages (600 mg/kg) group relative to the ETEC K88 group. These results indicate that ETEC K88 cause intestinal morphology impairment, which was revesed by dietary supplementation with phages. The protective effects of dietary phage supplementation on intestinal integrity were further supported by reduced serum DAO and D-lactate levels, along with upregulated jejunal ZO-1 protein expression in piglets from the ETEC K88 + phages group compared to the ETEC K88 group. Serum DAO and D-lactate levels are critical biomarkers for assessing intestinal barrier integrity [30]. ZO-1 is an critical scaffolding protein in tight junctions, functioning as a structural intermediary that connects the plasma membrane to the cytoskeletal framework [40].

Inflammation is a symptom of ETEC K88 infection and is associated with PWD in piglets [41]. Therefore, we evaluated the systemic and jejunal inflammatory responses of piglets to the experimental treatments. In this study, serum levels of LPS, IL-1β, IL-10, and TNF-α in piglets were higher in the ETEC K88 group than in the CON group, whereas these levels were lower in the ETEC K88 + phages group compared to the ETEC K88 group. These findings suggest that dietary phage supplementation alleviated the systemic inflammation induced by ETEC K88 infection. Furthermore, intestinal inflammation caused by ETEC K88 infection was also reduced by phage supplementation, as evidenced by the downregulated relative mRNA levels of *IL-1β* and *IL-6* in the jejunum of piglets from the ETEC K88 + phages group relative to the ETEC K88 group. The findings are consistent with our previous study, which demonstrated that phage administration alleviated *Escherichia coli*-induced diarrhea in weaned mice by modulating intestinal inflammation and gut microbiota [13]. To further investigate the underlying mechanism of intestinal inflammation, we measured jejunal protein expression related to the TLR4/NF-κB pathway in piglets. Compared to the CON group, jejunal protein expression of TLR4, p-IκBα, p-IκBα/IκBα, p-p65, and p-p65/p65 was upregulated in the ETEC K88 group. In contrast, the ETEC K88 + phages group showed downregulation of p-IκBα, p-IκBα/IκBα, and p-p65/p65 protein levels relative to the ETEC K88 group. Toll-like receptor 4 (TLR4) is a specific receptor for lipopolysaccharide from Gram-negative pathogenic bacteria, including *E. coli* [42]. Upon recognition of lipopolysaccharide by its specific receptor TLR4, the NF-κB signaling pathway is activated in the intestine of piglets [43]. Under normal physiological conditions, NF-κB remains in an inactive state in the cytoplasm due to its association with inhibitor κB (IκB), which binds to and suppresses NF-κB activity [44]. Phosphorylation of IκB is induced upon activation by the IKK complex, resulting in its dissociation from NF-κB and subsequent NF-κB activation [44]. The activated phosphorylated form of NF-κB translocates to the nucleus [45], where it induces the transcriptional activation of pro-inflammatory mediators such as IL-1β, IL-6, and TNF-α [44]. Therefore, dietary supplementation with phages inhibits the TLR4/NF-κB pathway and mitigates inflammation in piglets with ETEC K88 infection.

The gut microbiota constitutes a highly complex and stable ecosystem that fulfills a multitude of beneficial roles for the host, including the critical function of protecting against pathogenic colonization [46]. It is well-established that intestinal infections caused by ETEC K88 significantly influence the diversity and structural composition of the gut microbiota in piglets [47, 48]. In the present study, ETEC K88 infection decreased the Shannon index, while dietary phage supplementation increased the Shannon index in the fecal microbiota of ETEC K88-infected piglets. These findings suggest that phage supplementation can counteract the reduction in α-diversity of fecal microbiota induced by ETEC K88 infection. Regarding microbial composition of main dominant bacteria, compared to the CON group, the relative abundance of Proteobacteria, Enterobacteriaceae, and *Escherichia-Shigella* increased, while the relative abundance of Cyanobacteria decreased in the ETEC K88 group. The findings were validated through examination of the fecal microbiota utilizing LEfSe analysis. The phylum Proteobacteria is a microbial indicator of dysbiosis in the gut microbiota, with elevated abundances of Proteobacteria signifying an unstable microbial community (dysbiosis) and potentially serving as a diagnostic marker for disease [49]. In the current study, Spearman correlation analysis shows that the abundance of Proteobacteria exhibited a positive correlation with rectal temperature at both 12 and 24 h post-infection, as well as with serum TNF-α levels and jejunal *IL-1β* and *TNF-α* mRNA levels. The family Enterobacteriaceae and genus *Escherichia-Shigella*, both belonging to the phylum Proteobacteria, were reported to be significantly associated with inflammation [50, 51]. Indeed, the abundance of Enterobacteriaceae or *Escherichia-Shigella* was positively correlated with rectal temperature at 12 and 24 h post-infection, serum TNF-α level, and jejunal *IL-1β*, *IL-10*, and *TNF-α* mRNA levels. The findings indicate that ETEC K88 infection induced gut microbiota dysbiosis in piglets. However, dietary phage supplementation promoted gut microbiota eubiosis, as partially indicated by the decreased relative abundance of Proteobacteria and Enterobacteriaceae in the ETEC K88 + phages group compared to the ETEC K88 group. The relative abundance of Patescibacteria, Muribaculaceae, and *Subdoligranulum* was higher in the ETEC K88 + phages group compared to the ETEC K88 group. This finding further supports the role of dietary phage supplementation in promoting gut microbiota eubiosis, as these three bacterial taxa have been reported to confer host benefits [52–54]. Consistently, Spearman correlation analysis reveals that the abundance of Patescibacteria was positively correlated with jejunal villus height but negatively correlated with serum TNF-α level and jejunal *IL-1β* mRNA level. Besides, the abundance of Muribaculaceae was negatively correlated with rectal temperature at 24 h post-infection and jejunal *TNF-α* mRNA level. The correlation analysis confirms the role of altered microbiota in modulating diarrhea characteristics, intestinal integrity, and inflammatory responses in ETEC K88-infected piglets receiving phage-supplemented diets.

## Conclusions

Three ETEC K88-specific phages were successfully isolated, characterized, and lyophilized. Dietary supplementation at 600 mg/kg lyophilized phages reduced post-weaning diarrhoea by restoring intestinal barrier integrity, attenuating inflammation, and rebalancing gut microbiota. These results highlight phages as a promising strategy for controlling ETEC K88-associated PWD in weaned piglets in the post-antibiotic era.

## Supplementary Information

Additional file 1: Table S1 PCR primers for enterotoxin-producing genes of ETEC K88 (O141:K85, K88ab). Fig. S1 PCR results for enterotoxin-producing genes in ETEC K88 (O141:K85, K88ab). Fig. S2 Full uncropped blot images of Western Blot. Fig. S3 Full uncropped gel images of PCR products for enterotoxin-producing genes of ETEC K88 (O141:K85, K88ab).

## Data Availability

The raw genome data of the three phages were deposited in the GenBank database of the National Center for Biotechnology Information (NCBI) under the following accession numbers: BankIt2899308 EC-P1 (PQ657772), BankIt2895556 EC-P2 (PQ631072), and BankIt2899079 EC-P3 (PQ657771). The 16S raw sequences of fecal microbiota were submitted to the NCBI Sequence Read Archive under accession number PRJNA1290277.

## Abbreviations

ADFI: Average daily feed intake
ADG: Average daily gain
DAO: Diamine oxidase
ETEC: Enterotoxigenic *Escherichia coli*
FCR: Feed conversion ratio
IL-1β: Interleukin-1β
IL-6: Interleukin-6
IL-10: Interleukin-10
IκBα: NF-kappa-B inhibitor alpha
LB: Luria-Bertani
MOI: Multiplicity of infection
NF-κB: Nuclear factor kappa-B
PBS: Phosphate-buffered saline
PFU: Plaque forming unit
PWD: Post-weaning diarrhea
TLR4: Toll-like receptor 4
TNF-α: Tumor necrosis factor-α
ZO-1: Zonula occludens-1
