# Supplementary material for: Strategically isolated bacteriophages targeting ETEC K88 (F4) alleviate post-weaning diarrhea in piglets via modulation of gut microbiota and inflammatory responses
Source: J Anim Sci Biotechnol. 2026 Jan 18;17:11. doi: 10.1186/s40104-025-01322-6 (PMC12812250; doi:10.1186/s40104-025-01322-6)
Supplement: Supplementary file 1 — Additional file 1: Table S1 PCR primers for enterotoxin-producing genes of ETEC K88 (O141:K85, K88ab). Fig. S1 PCR results for enterotoxin-producing genes in ETEC K88 (O141:K85, K88ab). Fig. S2 Full uncropped blot images of Western Blot. Fig. S3 Full uncropped gel images of PCR products for enterotoxin-producing genes of ETEC K88 (O141:K85, K88ab). [file 40104_2025_1322_MOESM1_ESM.docx]

**Additional file 1**

**Table S1** PCR primers for enterotoxin-producing genes of ETEC K88 (O141:K85, K88ab)

| **Gene** | **Primer sequences (5´→3´)** | **Amplified fragment size, bp** |
| --- | --- | --- |
| *estA* | F: GAAACAACATGACGGGAGGT | 229 |
|  | R: GCACAGGCAGGATTACAACA |  |
| *estB* | F: CCTACAACGGGTGATTGACA | 480 |
|  | R: CCGTCTTGCGTTAGGACATT |  |
| *elt-Ⅰ* | F: GGTTTCTGCGTTAGGTGGAA | 605 |
|  | R: GGGACTTCGACCTGAAATGT |  |
| *elt-Ⅱ* | F: AGATATAATGATGGATATGTATC | 300 |
|  | R: TAACCCTCGAAATAAATCTC |  |

| 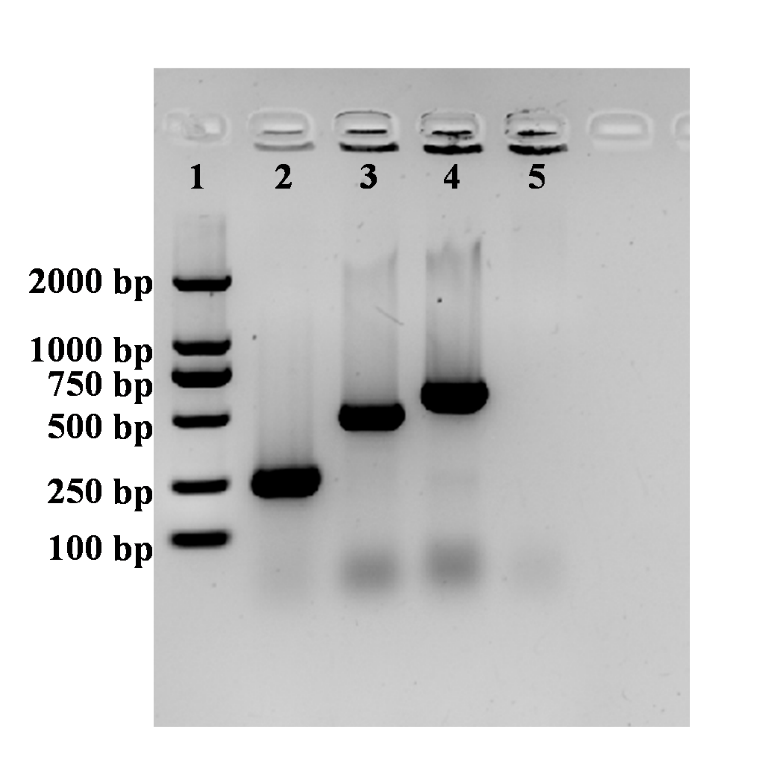 |
| --- |

**Fig. S1** PCR results for enterotoxin-producing genes in ETEC K88 (O141:K85, K88ab). Lane 1: DNA molecular weight marker; Lane 2: enterotoxin-producing *estA* gene; Lane 3: enterotoxin-producing *estB* gene; Lane 4: enterotoxin-producing *est-I* gene; Lane 5: enterotoxin-producing *est-II* gene. The PCR system was developed to identify enterotoxin genes (*estA*, *estB*, *elt-Ⅰ*, and *elt-Ⅱ*) in ETEC K88 (O141:K85, K88ab). Specific primers targeting the enterotoxin genes were designed as detailed in Table S1 and synthesized by Tsingke Corporation (Hunan, China)

| **A** | **B** | **C** |
| --- | --- | --- |
| 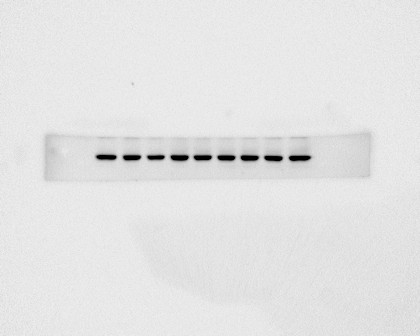 | 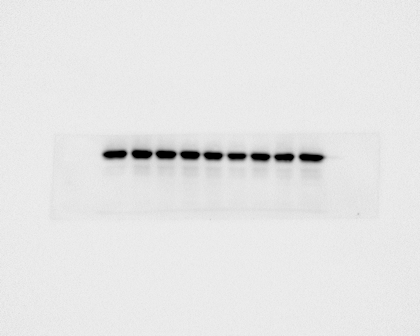 | 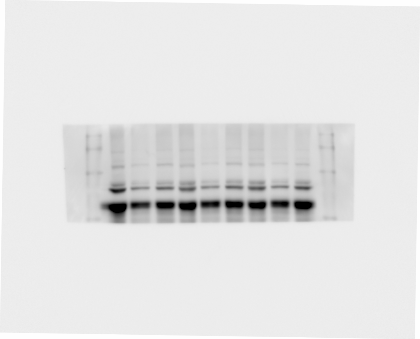 |
| **D** | **E** | **F** |
| 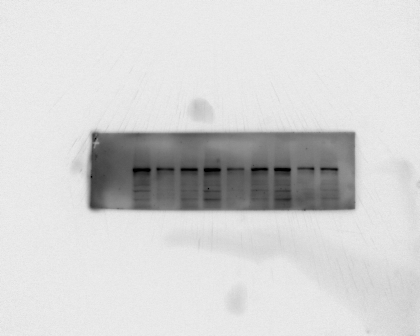 | 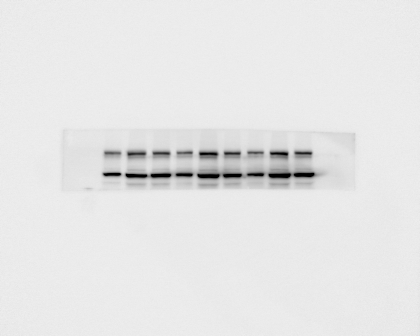 | 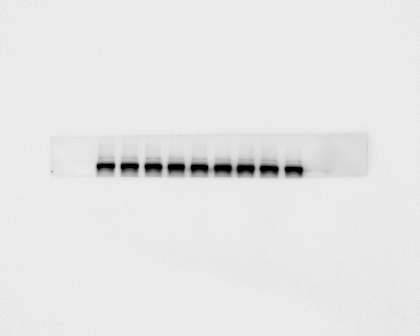 |
| **G** | **H** | **I** |
| 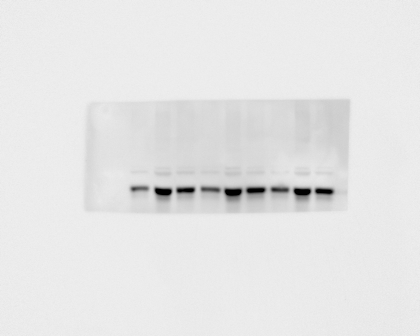 | 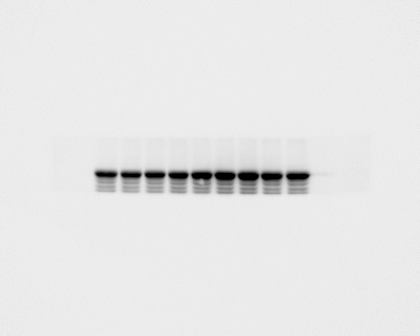 | 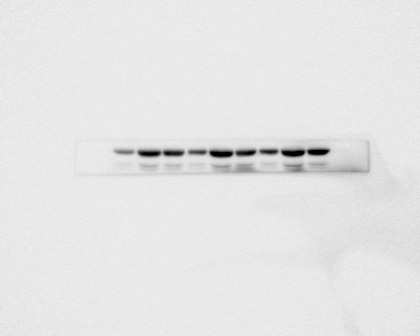 |

**Fig. S2** Full uncropped blot images of Western Blot. **A** β-actin. **B** Claudin-1. **C** Occludin. **D** ZO-1. **E** TLR-4. **F** IκBα. **G** p-IκBα. **H** p65. **I** p-p65

**
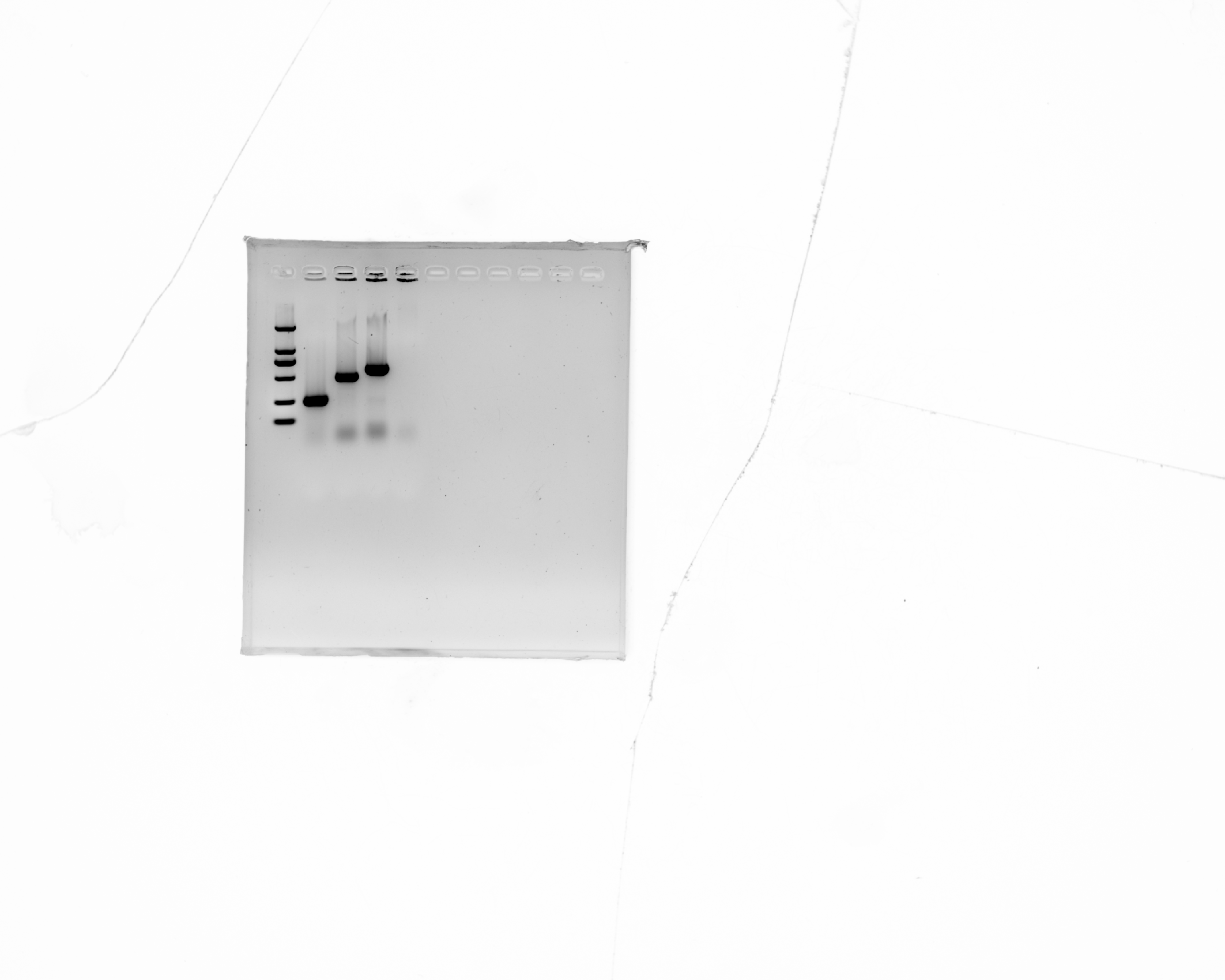
**

**Fig. S3** Full uncropped gel images of PCR products for enterotoxin-producing genes of ETEC K88 (O141:K85, K88ab)
